# Fabrication Methods and Luminescent Properties of ZnO Materials for Light-Emitting Diodes

**DOI:** 10.3390/ma3042218

**Published:** 2010-03-24

**Authors:** Ching-Ting Lee

**Affiliations:** Institute of Microelectronics, Department of Electrical Engineering, National Cheng Kung University, Tainan 701, Taiwan; E-Mail: ctlee@ee.ncku.edu.tw; Tel.: 886-6-2082368; Fax: 886-6-2082368

**Keywords:** ZnO thin film, fabrication methods, luminescent properties, defect states, conductive types, nanostructured ZnO, light-emitting diodes

## Abstract

Zinc oxide (ZnO) is a potential candidate material for optoelectronic applications, especially for blue to ultraviolet light emitting devices, due to its fundamental advantages, such as direct wide band gap of 3.37 eV, large exciton binding energy of 60 meV, and high optical gain of 320 cm^−1^ at room temperature. Its luminescent properties have been intensively investigated for samples, in the form of bulk, thin film, or nanostructure, prepared by various methods and doped with different impurities. In this paper, we first review briefly the recent progress in this field. Then a comprehensive summary of the research carried out in our laboratory on ZnO preparation and its luminescent properties, will be presented, in which the involved samples include ZnO films and nanorods prepared with different methods and doped with n-type or p-type impurities. The results of ZnO based LEDs will also be discussed.

## 1. Introduction

Zinc oxide (ZnO) based materials are potential candidates for optoelectronic applications, especially for blue to ultraviolet light emitting devices, due to its fundamental advantages, such as direct wide band gap 3.37 eV, large exciton binding energy 60 meV, and high optical gain 320 cm^−1^ at room temperature. Intensive research efforts have focused on ZnO and related devices for many decades. The quality of ZnO in the forms of both bulk and thin film has been improved substantially. The nanostructured ZnO materials have attracted great attention and various dimensions and shapes have been prepared. On the other hand, the luminescent properties of the ZnO related materials have been extensively investigated. The ZnO based LEDs of various device structures have been demonstrated. In addition, the exploration of new preparation methods is still in progress. 

In terms of the ZnO related investigations, we can say that great progress has been achieved, but there are still some obstacles to be overcome for realizing its wide optoelectronic applications, which remains a hot research direction. Large numbers of researchers are steadily working to explore this potential and promising field, and thousands of research papers are published each year. It is noted that various aspects of the investigation have been reviewed, such as in the comprehensive review papers [1,2]. In this paper, instead of a comprehensive review, we will emphasize the recent progress in a few selected topics we are interested in. For each topic, recent progress will be briefly reviewed and then the research activity conducted in our lab will be introduced in detail. 

This paper is arranged as follows. Section 2 is devoted to the progress in ZnO material preparation methods, especially the vapor cooling condensation method newly developed in our lab. Section 3 comments on the recent progress in the investigation on the luminescence of native defects, a widely observed phenomenon in various ZnO materials. The theoretical study of unintentional n-type conductivity is reviewed in Section 4. The exploration on p-type ZnO carried out in our lab is introduced in Section 5. The research of nanostructured ZnO materials, both on their preparation and properties, are summarized in Section 6. In Section 7, after a brief review, the recent progress in the research of ZnO based light emitting diodes conducted in our laboratory is presented, followed by a short summary in Section 8. 

## 2. Preparation Methods of ZnO Films

Growth of bulk ZnO crystals is mainly carried out by hydrothermal [3,4,5,6], vapor-phase [7,8,9], and melt growth [10,11] methods. However, the deposition of ZnO thin films has attracted more attention due to its application in optoelectronics and sensor devices. Many different techniques such as sputtering (dc sputtering, rf sputtering, and reactive sputtering) [12,13,14,15,16,17,18,19,20], pulsed laser deposition (PLD) [21,22], molecular beam epitaxy (MBE) [23,24,25,26], chemical vapor deposition (CVD) (including metal organic chemical vapor deposition ) [27,28,29,30,31], aqueous solution growth [32], spray pyrolysis [3,34], sol-gel method [35,36,37,38] and vapor cooling condensation method [39] have been used in the preparation of ZnO thin films as well as nanostructured ZnO. 

**Figure 1:**
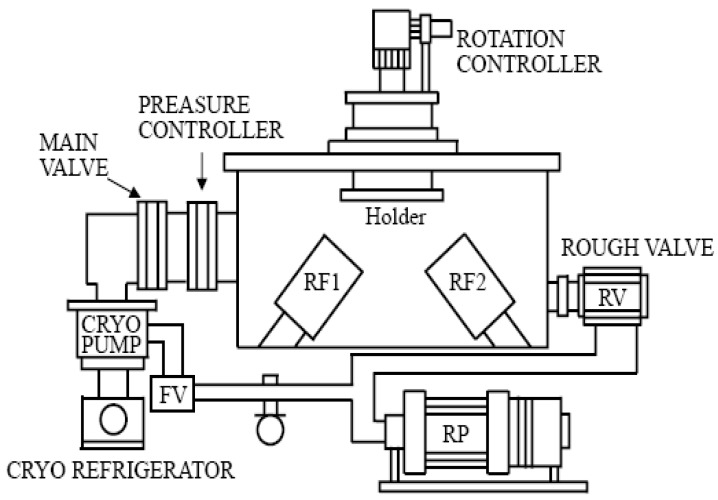
Schematic diagram of the co-sputtering system.

Rf sputtering is one of the most popular growth techniques for ZnO thin films. Although the earlier sputtered materials were polycrystalline or even amorphous, some research [16] has reported the high-quality single-crystal ZnO films deposited on sapphire (0001) by rf magnetron sputtering. They found that a high substrate temperature was essential to improve the crystal structure, but rf power had to be adjusted for the appropriate growth rate. We have extensively investigated the relationship between the crystal structure, doping concentration and properties of the sputtered films and the sputtering conditions [18,19,20]. Figure 1 shows a schematic of the rf magnetron co-sputtering system used in our lab. In this system [40], a dual rf power supply with synchronized phase is employed to control the rf power at the ZnO target and the dopant target, respectively. The substrate holder is rotating during the deposition to improve the uniformity of the thickness and the doping content of the deposited films. The substrate holder temperature can be controlled too. 

**Figure 2:**
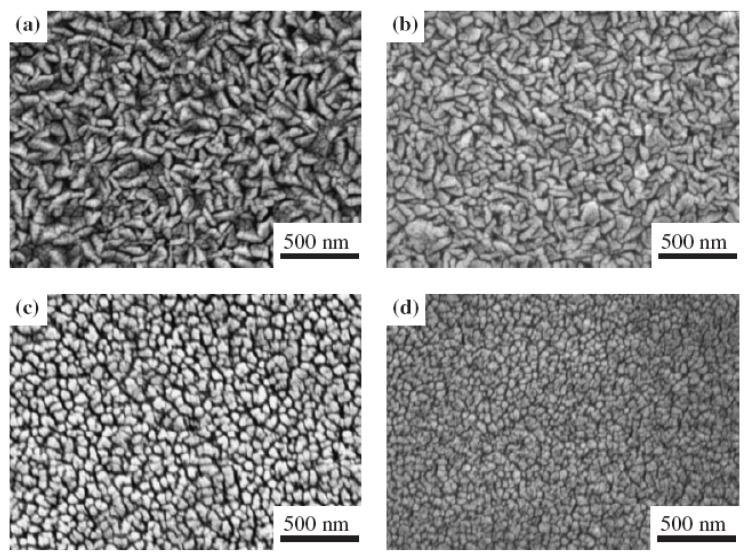
FE-SEM images of the (a) undoped ZnO and co-sputtered Al–ZnO films at theoretical Al atomic ratios of (b) 7.5, (c) 10, and (d) 12.5 at. %. Reprinted from [18] with permission.

Figure 2 shows the FE-SEM images of (a) undoped ZnO film and co-sputtered Al–ZnO films, with the rf power on ZnO target fixed and the power supplied to Al target to be (b) 26 W, (c) 40 W, and (d) 55 W [18]. In these conditions the corresponding theoretical Al atomic ratios [Al/(Al+ Zn) at %] in the co-sputtered ZnO films were evaluated to be (b) 7.5, (c) 10, and (d) 12.5 at % according to the individual deposition rates of the undoped ZnO and metallic Al films. All the SEM images show a grainy morphology. It was found that the grain size decreased as the Al atomic ratio in the co-sputtered film increased, indicating that the grain growth was inhibited by introducing Al into the ZnO matrix. The inhomogeneous wedge-like grains showed an evidence of lateral growth that resulted in the random growth orientation. The evolutions of the grain size and shape observed by FE-SEM coincided with the results derived from the XRD diffraction patterns.

**Figure 3:**
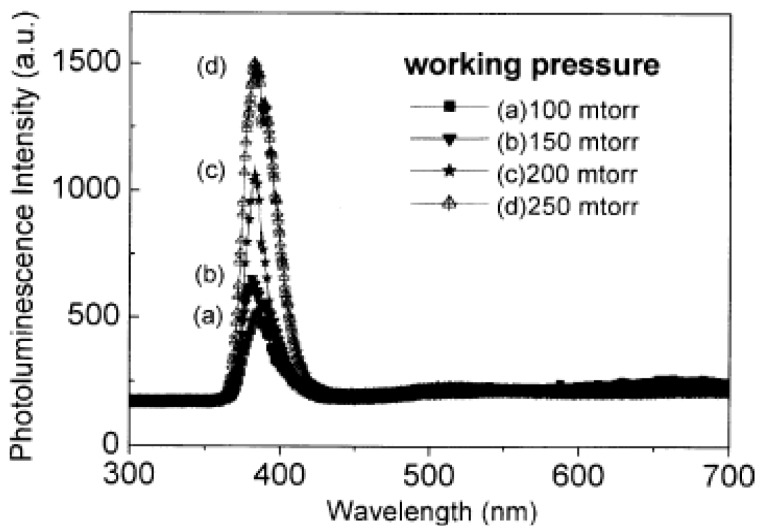
Photoluminescence spectra of ZnO films deposited under various working pressures. Reprinted from [19] with permission.

The effects of working pressures on the electronic and optical properties of undoped ZnO films deposited on Si substrates were studied [19]. It is found that the resistivity of the deposited ZnO films decreases with the working pressure, and the resistivity of 4.3 × 10^−^^3^ Ωcm can be obtained without post annealing (Table 1). The optical transmittance measurements gave a value above 90% at a wavelength longer than 430 nm and about 80% at the wavelength of 380nm for the deposited film. The time-resolved PL measurement showed that the carrier lifetime increases with the working pressure, indicating a reduction of nonradiative recombination rate. It can be attributed to the decrease of oxygen vacancies in the ZnO films deposited at a higher working pressure. This result is verified by the photoluminescence measurements (Figure 3), in which the UV PL intensity increases with the working pressure. Besides, with increasing the working pressure, the absorption coefficient decreased and the associated optical energy gap of ZnO thin films increased. The contents of sputtering gas are also important for the deposition, which will be discussed later.

**Table 1:** Resistivity, electron mobility, electron concentration and deposition rate of ZnO films deposited on Si substrates at 100W rf power and 20 sccm Ar flow rate with various working pressures. Reprinted from [19] with permission.

Working pressure (mTorr)	Property				
	Resistivity (×10^−3^ Ωcm)	Electron mobility (cm^2^ V^−1^s^−1^)	Electron concentration (1 cm^−3^)	Deposition rate (nm min^−1^)	Sample no.
100	44	1.75	8.04×10^19^	5.33	(a)
150	24	2.83	9.19×10^19^	3.96	(b)
200	8.9	6.33	1.10×10^20^	3.33	(c)
250	4.3	10.1	1.42×10^20^	3.10	(d)

**Figure 4:**
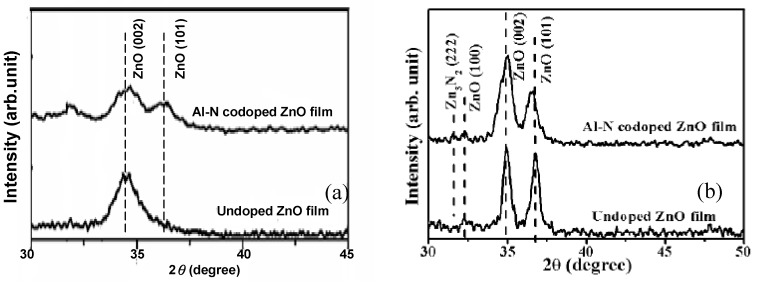
XPD patterns of undoped ZnO film and AlN codoped ZnO film (theoretical Al atomic ratio of 10%). (a) as-deposited (b) post-annealed at 400 °C under nitrogen ambient for 30 min. Reprinted from Ref. [20] with permission.

Figure 4 shows the XRD patterns of undoped ZnO and AlN codoped ZnO films, showing the effect of post-annealing [20]. An apparent diffraction peak of ZnO (002) phase was observed in the diffraction pattern of as-deposited undoped ZnO film, while the crystalline structure of as-deposited AlN codoped ZnO film was more disordered, as seen from Figure 4(a). However both the codoped and undoped ZnO films annealed at 400 °C under nitrogen ambient exhibited polycrystalline structures with the dominated diffraction peaks of ZnO (002) and (101) phases in the diffraction patterns. In addition, except for the ZnO-related diffraction peaks, a weak diffraction peak determined as Zn_3_N_2_ (222) was observed from the annealed Al–N codoped ZnO film diffraction pattern. The appearance of the zinc nitride phase was believed to be the nitrification reaction of the excess Zn and N atoms in the codoped films, indicating the excitation of the N ions after thermal annealing. *P*-type conductive behavior of AlN codoped ZnO was obtained after an additive post-annealing treatment at temperatures ranging from 400-600 °C under nitrogen ambient for 30 min, which will be discussed in Section 5. 

**Figure 5:**
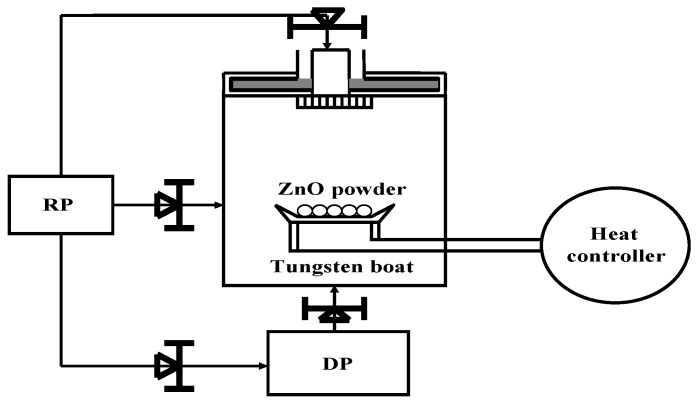
Setup of vapor cooling evaporation system.

We have developed a vapor cooling condensation method [39], with which high quality intrinsic ZnO (i-ZnO) films can be fabricated at low temperature. The schematic vapor cooling condensation system is shown in Figure 5.

**Figure 6:**
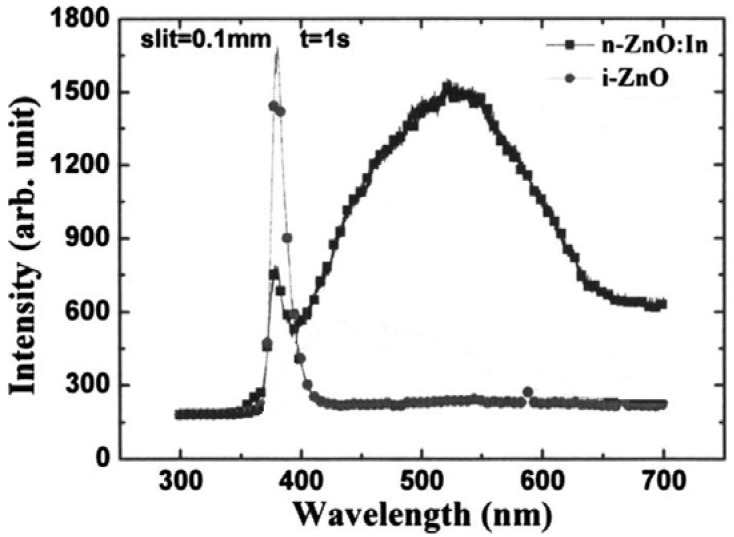
Room temperature PL spectra of the *i*-ZnO and *n*-ZnO:In films, excited at 325 nm. Reprinted from Ref. [39] with permission.

By heating the tungsten boat loaded with 0.85 g of ZnO powder, a 300 nm thick *i*-ZnO film was deposited on the substrate cooled by liquid nitrogen. An electron concentration of 7.6 × 10^15^ cm^−3^ and mobility of 2.1 cm^2^/Vs of the deposited *i*-ZnO film at room temperature were obtained. The room temperature PL spectrum of the resultant *i*-ZnO film is shown in Figure 6, which consists of a strong ultraviolet emission band centering at 382 nm with a full width at half maximum of 13 nm. The absence of visible emission implies a very low defect concentration owing to the low temperature growth. The system was also used for *n*-ZnO films deposition with pure ZnO and In as source materials. The electron concentration and mobility of the deposited ZnO:In films were 1.7 × 10^20^ cm^−3^ and 3.7 cm^2^/Vs, respectively. The PL spectrum of the *n*-ZnO:In films is also presented in Figure 6.

**Figure 7:**
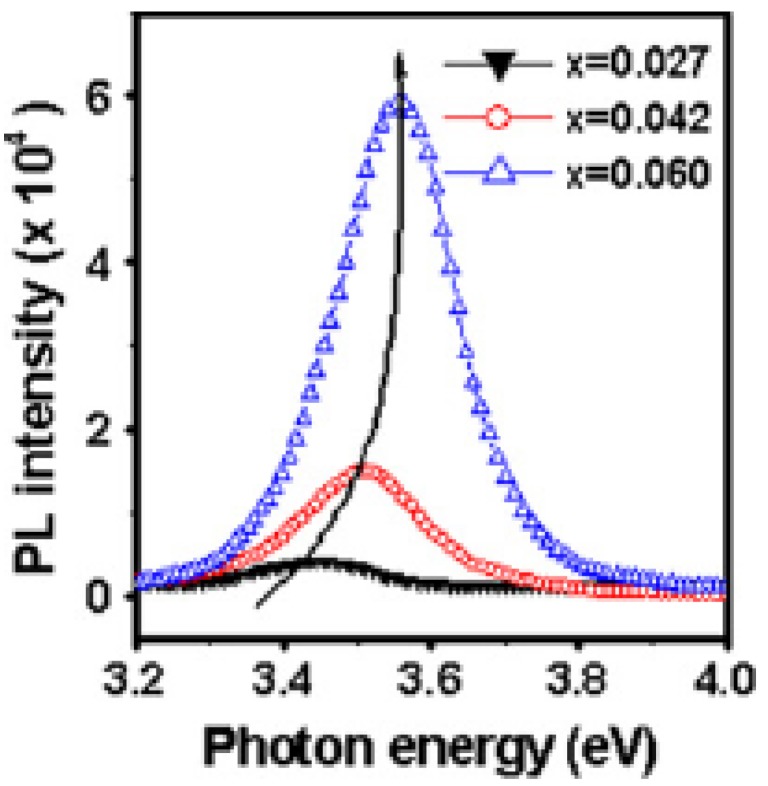
BEL spectra of the Zn_1__−_*_x_*Mg*_x_*O (*x* = 0*.*027, 0.042 and 0.060) films in the region from 3.2-3.9 eV. Reprinted from Ref. [38] with permission.

In order to realize modern devices, modulation of the bandgap of the material is required, which has been demonstrated by the development of Zn_1–x_Mg_x_O [26,41,42,43] and Zn_1–z_Be_z_O [44,45] alloys for the larger bandgap material and Zn_1–y_Cd_y_O alloy for the smaller bandgap material [46,47], showing a wide tuning range for ZnO-based materials. In our lab sol-gel method was used to perform the band gap engineering [37,38]. Zn_1__−_*_x_*Mg*_x_*O (x = 0.027, 0.042, and 0.060) films were prepared by the sol–gel method and spin coating technique, using Zn(CH_3_COO)_2_·H_2_O and Mg(CH_3_COO)_2_·H_2_O as start materials and methanol as solvent. After depositing by spin coating, the films were dried at 300 °C for 10 min on a hotplate to evaporate the solvent and remove organic residuals. The procedures from coating to drying were repeated many times. The films were finally annealed in air at 500 °C for 4 h. XRD profiles of the resultant Zn_1__−_*_x_*Mg*_x_*O films suggest the formation of wurtzite structure with a preferred *c*-axis orientation. And no evidence of MgO phase was seen, confirming the monophasic nature of these compositions. Figure 7 is the BEL (Band-edge luminescence) spectra of Zn_1__−_*_x_*Mg*_x_*O (*x* = 0*.*027, 0.042 and 0.060) films [38]. Obvious blue shift was observed when the content of Mg increased, which resulted from an increasing of the band gap.

## 3. Photoluminescence (PL) of Native Defects in ZnO

The origin of luminescent transitions is always one of central investigation topics for luminescent materials. Large amount of research efforts have been devoted to the luminescence properties of ZnO for more than half a century, including both intrinsic and extrinsic luminescence. However, in comparison with the study on the extrinsic luminescence of ZnO, the exact origin of its intrinsic luminescence due to native defects is still controversial. In this section, we will review the new progress obtained in recent years on the luminescence of native defects in ZnO, especially the progress in theoretical study of the defect states.

A typical low-temperature PL spectrum of the nominally undoped ZnO contains sharp and intense excitonic lines in the UV region of the optical spectrum, with one or more broad bands in the visible region. In spite of numerous reports on red, orange, yellow, and green broad bands, observed mostly at room temperature, their likely geneses are still speculative at this time. These bands are attributed to a variety of native defects such as oxygen vacancies *V_O_*, zinc vacancies *V_Zn_*, and oxygen interstitials *O_i_*, but very little is known about the properties of defects that cause various bands in the luminescence of undoped ZnO.

For example, the well-known green luminescence (GL) band centering at about 2.5 eV in undoped ZnO usually dominates the defect-related part of the PL spectrum. However, the nature of it remained controversial for decades. Özgür *et al.* [1] pointed out that while similar in position and width, these PL bands may actually be of different origins: the GL band with a characteristic fine structure is most likely related to the copper impurities [48], whereas the structureless GL band with nearly the same position and width may be related to native point defects such as *V_O_* or *V_Zn_*. Meanwhile, some works [49,50,51,52] speculated the oxygen vacancies, some works [53,54] speculated the zinc vacancies cause the GL band. 

Especially Leiter *et al.* [55,56] have identified *V*_*O*_ as the defect responsible for the structureless GL band in ZnO and demonstrated striking similarities of this defect to the anion vacancy in other ionic host crystals: BaO, SrO, CaO, and MgO (F centers). In their model the two-electron ground state of the neutral *V*_*O*_ is a diamagnetic singlet state, absorption of a photon transforms the system into a singlet excited state, followed by a nonradiative relaxation into the emissive, paramagnetic state (S=1) which can be detected by ODMR, then the optical cycle is closed by radiative recombination back to the S = 0 ground state. In a recent work [57], Hofmann *et al.* concluded that the PL and DLTS (deep level transient spectroscopy) experimental results suggested a correlation between the GL and a donor level 530 meV below the conduction band, which is attributed to the $V_{O}^{0/++}$ transition of oxygen vacancies. 

TThe GL bands were often found in observed PL spectra of samples prepared in our lab and they usually were attributed to oxygen vacancy defects. For example, a green-band emission was observed both for the undoped ZnO film and the p-type AlN codoped ZnO films [58], and the intensities were smaller for the latter, which implies that less oxygen vacancies existed in them. However, in a work on ZnO-on-GaN heterostructures grown using the vapor cooling condensation system [39], compared to undoped ZnO, a remarkable broad green emission band in ZnO:In films was observed, and it was considered to be induced by the oxygen vacancies. Moreover, in our work on ultraviolet (UV) emission of In-doped ZnO nanodisks grown by carbothermal reduction at 1000 °C [59], air-cooled ZnO nanodisks showed a strong green emission, while furnace cooling in conjunction with introducing O_2_, around 1.0%, into flowing Ar during the growth significantly enhanced the growth rate and UV emission of ZnO nanodisks (while the green emission was significantly suppressed). The causes were attributed to the reduction of oxygen vacancies and surface defects. Besides, for the Zn_1__−_*_x_*Mg*_x_*O films prepared by the sol–gel technique [38], a GL was observed and its intensity decreased (while the intensity of the band-edge luminescence was seen to increase) with the increase of Mg content, which was partly attributed to a decrease in the number of *V_O_*-related defects. 

However, deferent results were also reported. Recently Kappers *et al.* [60] studied ZnO crystals grown by the seeded chemical vapor transport method. The samples were irradiated at room temperature with 1.5 MeV electrons to create oxygen and zinc vacancies, and then characterized using optical absorption, PL, and electron paramagnetic resonance (EPR). It was found that no correlation existed between the green emission and the presence of oxygen and/or zinc vacancies. Similarly, Vlasenko and Watkins [61] also found that electron irradiation produced O vacancies and other defects in ZnO, leading to a reduction in GL and an increase in PL bands near 600 and 700 nm. These works questioned the assumption that GL is related to *V_O_*. In addition, we noticed that the formation energy of zinc vacancies is as high as 4 eV in n-type ZnO [62], which implies that the concentration of *V_Zn_* in ZnO would be too low to cause the observed green band in the PL spectrum. So are the other native defects, for example, zinc interstitials [63] and oxide antisite defects [64,65], as have been suggested previously.

**Figure 8:**
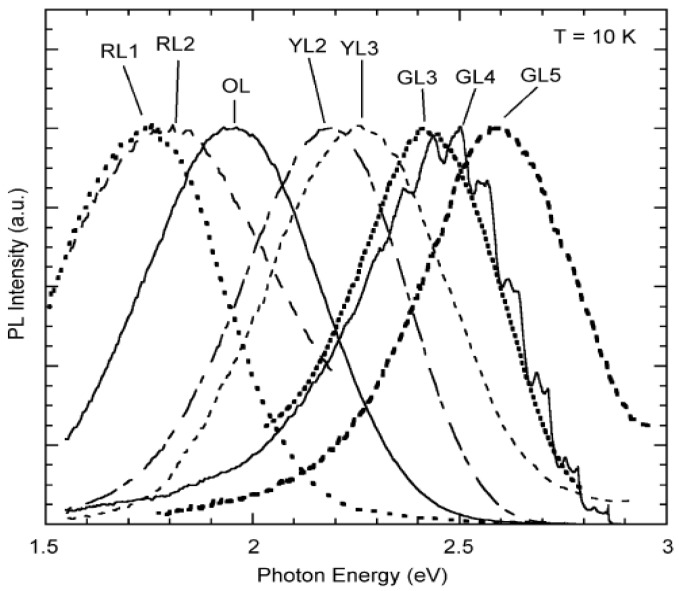
Selected PL bands observed at 10 K in ZnO. Intensity is normalized at band maxima. Reprinted from Ref. [66] with permission.

Recently, Reshchikov *et al.* [66] systematically investigated the PL of defects in ZnO. They analyzed carefully the PL spectra obtained in wide ranges of excitation power densities and sample temperatures, as well as the spectra at various time delays after a pulsed excitation. They resolved a number of PL bands in the visible region of the PL spectrum, as seen in Figure 8 and Table 2 (some bands could not be observed at either 10 or 300 K) in which E_A_ is the activation energy, Tcr is the critical temperature above which the quenching begins, both are quantities describing quenching behavior of the PL band.

**Table 2:** Main characteristics of visible PL bands in ZnO. Reprinted from Ref. [66] with permission.

PL band	Peak position (eV)		E_A_(meV)	T_cr_(K)
	10 K	300 K		
RL1	1.75	~1.75	15	30
RL2	1.8	~1.9	~150	200
OL	1.95	2.12	500	240
YL1	2.15	−	450	240
YL2	2.19	2.2	85	100
YL3	2.25	−	500	200
GL1	−	~2.3	~500	260
GL2	2.38	−	35	40
GL3	2.42	2.44	400	260
GL4(Cu)	2.47	2.47	~80	50
GL5	2.6	−	120	100

Based on the careful analyses of the spectra they reached the following conclusion. The majority of the defects remained unidentified and transitions responsible for particular bands need to be verified. The most recognizable PL bands are the OL band peaking at 1.96 eV at 10 K (assigned to transitions from shallow donors to a deep acceptor level about 0.5–0.6 eV above the valence band [67]) and the Cu-related GL4 band with the characteristic fine structure.

Theoretically, there were also some first-principles calculations on electronic structure of defects in ZnO [68,69] based mainly on the local density functional approximation (LDA) or added with various corrections (LDA+*U*, LDA/GGA, *etc.*). However, these works always gave the fundamental band gap value E_g _< 1 eV (compared to experiment value 3.4 eV) for several tens of years, therefore the defect levels within the gap can only be estimated by means of various empirical correction. Recently, based on the hybrid density functional computation program, a few authors [62,70,71] have made *ab initio* calculation of the band gap, atomic and electronic structure (especially of the defects) for ZnO. Their work gave correct band gap (E_g_ = 3.4 eV), and so the calculated results for defect states and levels within the band gap are much more reliable. 

To understand PL related native defect states in ZnO, Hu and Pan [71] studied vacancies $V_{O}^{q}$, $V_{Zn}^{q}$, and self-interstitials $Zn_{i,\text{o}}^{q}$, $O_{i,\text{o}}^{q}$ (occupying octahedral sites) with q = 0,+1or −1,+2 or −2. As part of the results, their table 1 listed all the calculated single-electronic energy levels (Kohn-Sham levels) at the Gama point of the Brillouin zone within the band gap of a modeling defect-crystal (MDC). In the calculation, they used a 72-atom supercell containing one isolated native defect of above kinds and the atom-configuration was completely relaxed. Their calculation is spin-polarized, namely, the same orbital state with spin-up or spin-down has different energy generally. This is obvious for supercell of odd-number electrons, in which the number of spin-up electrons must be different from the number of spin-down electrons, like the one containing defect with q = +1 or −1, since the exchange potential in Kohn-Sham equation is different for -up or -down single electron. However, for the $V_{Zn}^{0}$-containing MDC here, the calculated result is also spin-polarized. As pointed out by C.H. Patterson [70], the neutral Zn vacancy contains two “dangling holes”. It is noted that the hole-orbitals (unoccupied electron-orbitals) mainly consist of 2p orbitals of the four oxygen atoms around the vacancy. The “total-energy minimization” produces a spin-split single-electron energy level scheme where spin-up levels descend and are totally occupied and only two high-energy spin-down orbitals are left to be empty. Usually each MDC has several defect-states, the number of defect-levels within the band gap and the total number of electrons occupying each level can be different from each other. Therefore, the positions of the Fermi levels and the level-occupation under temperature T = 0 are also different for different MDC. 

**Figure 9:**
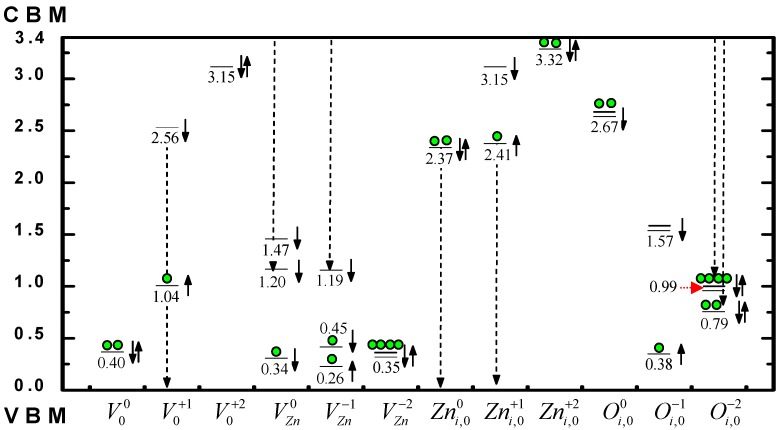
Calculated energy levels of native defects in the band gap of ZnO. Based on table 1 of Ref. [71].

For the reader’s convenience, in Figure 9 we plot an energy level diagram based on the data in Table 1 of their paper [71].

In Figure 9, the arrows “↑”, “↓”, and “↑↓” standing next to each level indicate that the corresponding levels are “spin up”, “spin down”, and “spin degeneration”, respectively. The double line “═” represents the doubly degenerate orbital. The solid sphere “●” represents an electron that occupies the level beneath it. The figure represents the occupation scheme under the temperature T = 0 for the ground state of the studied system containing the defect presented explicitly at the bottom of the figure. The dashed vertical arrow lines show the possible transitions corresponding to the green luminescence. The transitions conduction-band minimum (CBM)→1.47 eV ($V_{Zn}^{0}$) and CBM→1.57 eV ($O_{i,o}^{-1}$) correspond probably to the orange and red luminescence, respectively.

If only the peak positions of the PL bands are concerned, by comparing Table 2 with the figure shown above, we can make the following assignment:

RL1: CBM ---> 1.57 eV of $O_{i,\text{o}}^{-1}$
OL: CBM ---> 1.47 eV of $V_{Zn}^{0}$
YL2: CBM ---> 1.20 eV of $V_{Zn}^{0}$
or CBM ---> 1.19 eV of $V_{Zn}^{-1}$
GL2: 2.37 eV of $Zn_{i,\text{o}}^{0}$ ---> valence-band maximum (VBM)
GL3: 2.41 eV of $Zn_{i,\text{o}}^{+1}$ ---> VBM
or CBM ---> 0.99 eV of $O_{i,\text{o}}^{-2}$
GL5: CBM ---> 0.79 eV of $O_{i,\text{o}}^{-2}$
or 2.56 eV of $V_{O}^{+}$ ---> VBM

From the calculated results, they derived the conclusion that both the vacancies and interstitials of O and Zn contribute to the green PL (2.2–2.6 eV) observed in various experiments.

It should be noted that the electron-phonon (or electron-lattice) interaction may be strong for localized electronic states of defects. That is, the relaxed atom-configurations of different charge states of a defect are, in general, different from each other. The defect *V_O_* in ZnO gives a typical example, for which the different charge states have very different relaxed atom-configurations. For the neutral defect $V_{O}^{0}$, the four Zn neighbors move inward, with the distances between these Zn atoms contracting by about $\Delta_{0}=-$9% (or −12% [72]) relative to that in perfect ZnO, while for the charged $V_{O}^{2+}$, the distances expanding by about $\Delta_{2}=$ 19% (or 23% [72]). The origin of these large lattice relaxations can be interpreted by the change in electron distribution in different charge states of *V*_O_. The removal of an oxygen atom from the lattice breaks four bonds. The four “dangling bonds” on the surrounding Zn atoms combine to form a fully symmetric single-electron state *a*_1_ of energy in the band gap (and three almost degenerate states located above the CBM). In the neutral charge state, the *a*_1_ state is occupied by two electrons. The energy of the state is lowered as the four Zn atoms approach each other. At the same time, the Zn-O bonds are stretched. Finally the gain in electronic energy balances the cost to stretch the Zn–O bonds surrounding the vacancy at the corresponding equilibrium configuration, and the resulting *a*_1_ state lies in the gap near the top of the valence band. In the $V_{O}^{+}$ charge state, the *a*_1_ state is occupied by one electron. In this case, the competition of the electronic energy with the strain energy results in the four Zn atoms displacing slightly outward ($\Delta_{1}≅$ 2%), correspondingly the *a*_1_ state moves towards the middle of the band gap. In the $V_{O}^{2+}$ configuration, the *a*_1_ state is empty, which leads the four Zn atoms strongly relaxed outward, and the empty *a*_1_ state lies near and below the conduction band edge.

**Figure 10:**
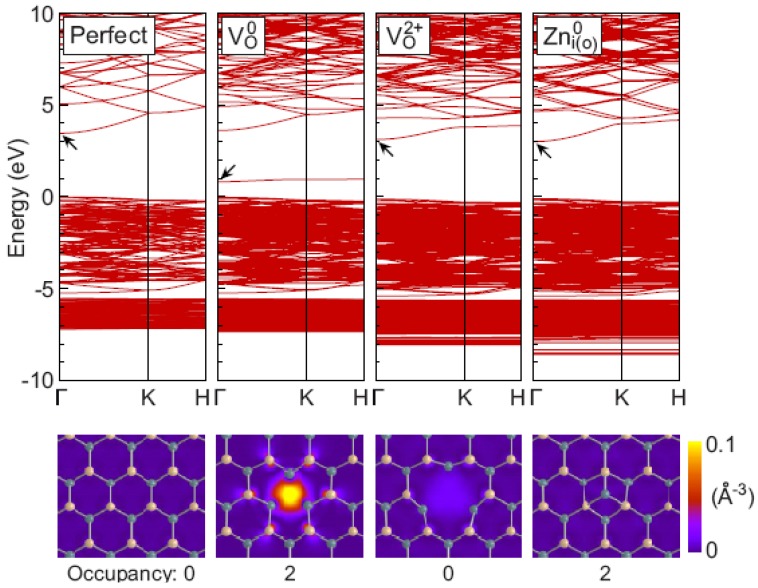
Band structure for bulk ZnO and defects $V_{O}^{0}$, $V_{O}^{2+}$, $Zn_{i}^{0}$ contained ones, which was obtained using the hybrid functional. Shown below are the squared wave functions of the states designated by the arrows in the corresponding band diagram, which are plotted for the middle of the (0001) Zn and O planes adjacent to the defects. The projected Zn and O atom positions are denoted with green (dark) and yellow (light) circles, respectively. Reprinted from Ref. [62] with permission.

Recently, refined results have been reported, in which Oba *et al.* [62] executed a first-principles calculation of the electronic structures of both charged and uncharged supercells containing 192 atoms by hybrid functional approach combining with careful finite-size corrections. The resultant band structures for perfect bulk ZnO and defect ($V_{O}^{0}$, $V_{O}^{2+}$, $Zn_{i}^{0}$) contained ZnO are presented in Figure 10.

As for the relaxation of atomic configuration around defects, they found $\Delta_{0}=-$10% and $\Delta_{2}=$ 23% for $V_{O}$, not very different from the values given above. In their work, the calculation errors due to the spurious electrostatic interactions between defects in these finite-sized supercells were corrected by introducing the energy terms of $L^{-1},L^{-3},$ … dependences (where *L* is the average interdefect distance), among which the $L^{-1}$ term corresponds to the Madelung energy for an array of point charges $q_{c}$ in an effective medium. It is noteworthy that, based on their calculation results, in contrast to the localized nature of the defect ($V_{O}$) states, the highest occupied state of $Zn_{i}^{0}$ is delocalized and comparable in energy position to the bottom of the conduction band of the perfect crystal. Due to this delocalized character, the removal of the electrons, *i.e.*, changing the charge state to + or 2+, does not alter the band structure. Furthermore, the filling of this delocalized conduction-band-like states does not effectively screen the charge of the point defect, leaving a long-range Madelung interaction (with $q_{c}$ = 2) between the point defects even in case of the neutral supercells. So does for the defects $Zn_{i}^{+},Zn_{O}^{0},Zn_{O}^{\text{+}}$ (similarly for shallow impurity defects $H_{i}^{0},H_{O}^{0}$ both of them have $q_{c}$ = 1).

However, it should be pointed out that the above calculation is based on a single electron approximation, in which even roughly from the point of view of Hartree approximation, the Kohn-Sham energy states are states of a single electron moving in an averaged field induced by interaction between electrons in a system. It is often to regard simply an optical transition as a transition of one electron between two Kohn-Sham levels of the system, calculating the difference between these levels as the transition energy. Actually, the optical transition energy of a multi-electron system is the difference of the total energies of the initial and final multi-electron states. The total energy of a multi-electron state contains the interaction between the electrons that occupy different single electron (Kohn-Sham) levels. Therefore the transition energy is generally different from the difference between the (transition-involved) two single electron energy levels within a single set of Kohn-Sham levels. In addition, different states of a multi-electronic system, in general, have different single-electron (Kohn-Sham) energy levels due to the different averaged field. In other words, the energies of all other electrons, and the interaction energy between them, are also changed after the studied one-electron optical transition, especially for transitions between different charge states of a localized multi-electron system as that in the case of defects. Then for properly describing the defect-related optical transition, a so-called optical transition level (see Section 4 for detail), an effective single electron energy level pertinent to the studied defect-related optical transition, has to be introduced into the band energy diagram instead of Kohn-Sham levels. Determination of the transition level is of great interest, but has not yet been systematically calculated as we know. Obviously the expected calculation will greatly push forward the study on the PL of defects in ZnO.

## 4. Theoretical Study of Unintentional N-Type Conductivity

The electronic properties of ZnO were traditionally explained by invoking intrinsic defects - the native and unintentionally introduced point defects in the past decades of years. In particular, the frequently observed unintentional *n*-type conductivity was often attributed to oxygen vacancies [73,74]. However, previous theoretical works related the defect $V_{O}$ with rather deep levels below CBM [72,75], indicating that further exploration of the origin of the unintentional n-type conductivity of ZnO has to be conducted. 

Recently, the native defects and the hydrogen impurity in ZnO were reinvestigated by Oba *et al.* [62], in which they calculated formation energy (total-energy-difference between defect and perfect systems) and the related transition levels for each kind of defects. The results were self-consistent and well consistent with the experimental ones. 

Here, we first give a brief introduction to the concept of the transition level. The transition level is related to the total energies of the initial and final defect states of a particular type of transition [76]. As an example, we consider the transition $V_{O}^{0}\to V_{O}^{+}+e_{m}$, where $e_{m}$ is an ionized electron staying at some single electron state of energy $E^{\prime }(e_{m})$. The total energy of the initial and final states of the transition of the multi-electron system considered here will be $E(V_{O}^{0})$ and $E(V_{O}^{+})+E^{\prime }(e_{m})$, respectively. Considering the ionized electron $e_{m}$ is independent of the defect V_O_, we can regard the transition in consideration as the transition of this particular electron $e_{m}$ by introducing an effective single electron energy level, so called transition level ε(0/+), namely, the transition can be expressed as $\varepsilon (0/+)\to E^{\prime }(e_{m})$ of the electron $e_{m}$. The level ε(0/+) is usually defined relative to the valence band top $E_{V}$ of the bulk ZnO and can be evaluated as follows. When the electron *e_m_* occupies the transition level, *i.e.* its energy ${E^{\prime }}_{\varepsilon }(e_{m})=\varepsilon (0/+)+E_{V}$, the equation $E(V_{O}^{0})=E(V_{O}^{+})+{E^{\prime }}_{\varepsilon }(e_{m})\;$ holds. From the equation we can get $\varepsilon (0/+)={E^{\prime }}_{\varepsilon }(e_{m})-E_{V}\text{=[}E(V_{O}^{0})-E(V_{O}^{+})]-E_{V}$. It can be clearly seen that the transition level contains the contribution of both multi-electron system $V_{O}^{0}$ and $V_{O}^{+}$. For the example discussed here the transition level ε(0/+) is positioned within the band gap. In general, the independent electron $e_{m}$ may sit at a single electron level of energy $E^{\prime }(e_{m})=E(e_{m})+E_{V}$, and then the excitation (ionization) energy of the system relative to the charge state $V_{O}^{0}$, $\left[E(V_{O}^{+})+E^{\prime }(e_{m})]-E(V_{O}^{0}\right)$, can be determined to be $E(e_{m})-\varepsilon (0/+)$ [where both $E(e_{m})$ and ε(0/+) are measured relative to VBM]. In particular, for the *e_m_* at CBM ($E(e_{m})=E_{g}$), the excitation energy is just $E_{g}-\varepsilon (0/+)$. For the reverse transition $V_{O}^{+}+e_{m}\to V_{O}^{0}$, similar discussion can be derived.

**Figure 11:**
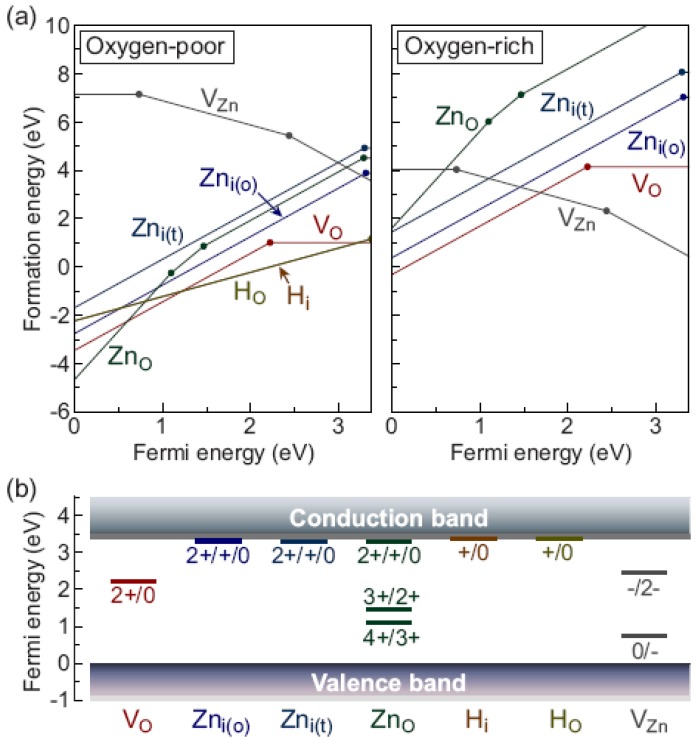
(a) Defect formation energies as a function of the Fermi energy at the oxygen-poor and oxygen-rich limits, (b) Defect thermodynamic transition levels. Reprinted from Ref. [62] with permission.

Now we can discuss the calculated results of Oba *et al.* Figure 11(a) [62] presents the formation energies as a function of the Fermi energy. The Fermi energy here is taken as the energy (chemical potential) of the reservoir from (in) which electrons are removed (placed) to form a charged defect [76], which is just the $E^{\prime }(e_{m})$ in the case mentioned above. Usually the range of the Fermi energy is chosen to be in the range between the calculated valence-band maximum (0 eV) and conduction-band minimum CBM (3.4 eV for bulk ZnO [62]). The formation energy of a defect in various charge states, say, $V_{O}^{+}$, contains the energy of the ionized electron, $e_{m}$ and hence varies with the Fermi energy. In Figure 11(a), for each kind of defect, only the charge states that are energetically most favorable at a given Fermi energy are shown. The Fermi energies, at which the slopes change (where, for example, the formation energy of $V_{O}^{0}$ equals the formation energy of the defect state $V_{O}^{2+}$.), indicating happening of transition between the two charge states of the defect, correspond to the positions of thermodynamic transition levels (because the calculated total energy for each charge state corresponded to the completely relaxed atom-configuration). The resultant transition levels are depicted in Figure 11(b) together with the relevant charge states.

Notably, among the native donorlike defects, *V*_*O*_ shows the lowest formation energy under most conditions. The formation energy for the neutral state $V_{O}^{0}$ is only 1.0 eV at the oxygen-poor limit, which is low enough to account for the observed nonstoichiometry: e.g., 190 ppm for the specimen treated at 1373 K. 

Oba *et al.* [62] confirmed that for defect *V*_*O*_, ε (2+/+) lies above ε (+/0) with U= ε(+/0)−ε(2+/+)=−0.7 eV (The defect is called a negative-*U* center). As the Fermi level moves from zero upward, the thermodynamic charge-state transition is thus directly from the 2+ to the 0 charge state, since the formation energy of $V_{O}^{+}$ is higher than that of $V_{O}^{0}$ and $V_{O}^{2+}$ for all values of *E_F_*, so the charge state $V_{O}^{+}$ is unstable for any position of the Fermi level. This negative-*U* behavior is typically related to unusually large local lattice relaxations that stabilize particular charge states mentioned above, since these large relaxations significantly reduce the formation energies of $V_{O}^{2+}$ and $V_{O}^{0}$ relative to $V_{O}^{+}$. At the same time they provide a thermodynamic transition level (2+/0) of *V_O_* at 1.2 eV below the CBM, very close to the convergence value 1.0 eV obtained by all methods that predict sufficiently large band gaps. 

The zinc interstitials, *Zn_i_*_(o)_ and *Zn_i_*_(t)_, have transition levels located at 0.05 and 0.1 eV below the CBM, respectively. These levels are regarded here as the transition levels among the three charge states, (2+/+/0), because the difference between the (2+/+) and (+/0) transition levels is comparable to the accuracy of the calculations. The possession of the (2+/+/0) transition levels near the CBM indicates that the zinc interstitials are single or double shallow donors, which is consistent with the experimental report (donor energy: 0.03 eV [77]). However, in these cases the formation energies are as high as 4 and 5 eV even at the oxygen-poor limit. Therefore, the zinc interstitials are unlikely to form with a substantial concentration in *n*-type ZnO.

$Zn_{O}$ also has a high formation energy under *n*-type conditions. It has a shallow (2+/+/0) transition level and two other levels (4+/3+) and (3+/2+) located below the middle of the band gap. 

Under the oxygen-poor condition and for a low Fermi energy, formation energy of $Zn_{O}$ is negative. It is the same for the other donorlike defects. A strong compensation of holes is therefore expected in ZnO grown under oxygen-poor conditions.

For the hydrogen impurity, both *H_i_* and *H_O_* show (+/0) transition levels locating nearly on the CBM, as reported in all the studies. The formation energies are 1.2 eV or lower, depending on the Fermi energy, which are close to an experimental estimate of 0.8 eV [78] and as low as that of *V_O_* at the hydrogen-rich (oxygen-poor) limit. Therefore, their results support the proposed role as a shallow donor for the hydrogen impurity [79].

Since *V_Zn_* has high formation energy (between 3.5 and 7.1 eV), it is not expected to exert significant effects on the composition and carrier concentration, therefore a strong preference for the donorlike defects over acceptorlike *V_Zn_* under oxygen-poor conditions is found. This tendency is consistent with experimental observation of the nonstoichiometric and *n*-type behavior of ZnO, but has not been reproduced by the previous calculations other than applying *a posteriori* band gap corrections to the LDA/GGA, as suggested in Ref. [75].

In summary, the recent theoretical work provides a clear picture of the defect energetics: among the donorlike defects, *V*_*O*_ as a deep donor, and *H_i_* and *H_O_* as shallow donors, are likely to form with a substantial concentration in *n*-type ZnO. *Zn_i_* and $Zn_{O}$ are shallow donors but their formation is energetically much less favorable. A strong preference for *V_O_*, *H_i_*, and *H_O_* over acceptorlike *V_Zn_* is expected under oxygen-poor conditions. It is therefore suggested that *V_O_* (and also *H_O_*, which can be regarded as a complex of *V_O_* and *H_i_*) should dominantly contribute to nonstoichiometry, and the hydrogen impurities, *H_i_* and *H_O_*, and/or metastable *V_O_* with a shallow donor state (see below) can effectively act as donors, both without significant compensation by *V_Zn_*. May be, this work constitutes a major step forward in modeling the complex behavior of semiconductors. To reach definite conclusion, further investigation, both theoretical and experimental investigations have to be conducted.

Now we turn to the discussion about the metastable *V*_*O*_ shallow donor state mentioned above. S. Lany and A. Zunger [80] pointed out that at the defects *V*_*O*_ in ZnO can take place a light-induced transition $V_{O}^{0}$*→*$V_{O}^{2+}$ + 2*e*, which results in metastable configuration change from nonconducting to conducting, constituting persistent electron photoconductivity (*n*-type PPC). Their model can be described as follows: first, both electrons occupy the deep and nonconducting *a*_1_-symmetry defect localized state (DLS) of the ground charge state $V_{O}^{0}$ (*d*_Zn-Zn_ = 3.0 Å). An optical excitation of energy 2.83 eV makes the $V_{O}^{0}$ ground state transformed to the $V_{O}^{+}$ + *e* excited state. This transition creates $V_{O}^{+}$ (*d*_Zn-Zn_ = 3.2 Å) having a singly occupied DLS within the band gap, which is observed in EPR (electron paramagnetic resonance) experiments under illumination [81,82]. The second excitation $V_{O}^{+}$*→*$V_{O}^{2+}$ + *e* of excitation energy 2.4 eV creates the charge state $V_{O}^{2+}$ with equilibrium *d*_Zn-Zn_ = 4.0 Å. Accompanying this ionization and large outward relaxation, the *a*_1_ DLS moves upward, becoming an unoccupied resonance state in the conduction band at (*E*_C_ + 0.4) eV. At the same time, the ionized $V_{O}^{2+}$ creates a perturbed-host state (PHS) below the CBM by a long-range and screened Coulomb potential, which is a delocalized, hydrogen-like shallow state. Consequently, the photoexcited electrons occupy the lower energy PHS (rather than the DLS), thus a conducting configuration constituting *n*-type PPC is established. This light-induced configuration is metastable against the depopulation of electrons from the PHS into the deep ground state with small *d*_Zn-Zn_ across an energy barrier. 

**Figure 12:**
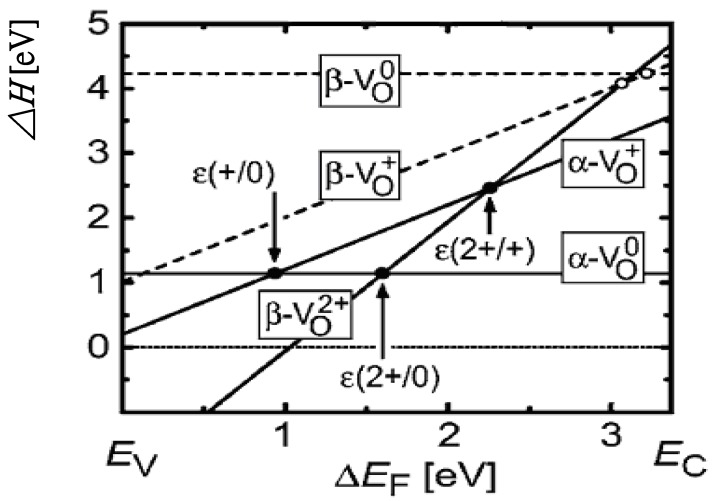
Formation energies of the different charge states of *V_O_* in ZnO. Solid lines and closed circles show the formation and transition energies of the equilibrium stable states, respectively. Dashed lines and open circles show the formation and transition energies in the light-induced metastable configuration. Reprinted from Ref. [80] with permission.

Figure 12 shows the formation energies of the light-induced metastable configuration of $V_{O}^{0}$ and $V_{O}^{+}$ (dashed lines) as a function of the Fermi energy *E*_F_, together with the corresponding formation energies in the respective equilibrium stable configuration (solid lines). The transition energies in the metastable configuration (open circles) are close to the CBM, so that this configuration is conductive. 

One point to be noted is that there are two kinds of transition levels $\varepsilon_{th}(q/q^{\prime })$ and $\varepsilon_{op}(q/q^{\prime })$, the thermodynamic and optical ones. The former is used for describing thermodynamic equilibrium processes and so is defined as the difference of the total (or formation) energies of two charge states (thermodynamic equilibrium states) of a same kind of defect, which in it’s definition are corresponding to the respective relaxed atomic configuration, and mostly have been calculated for ZnO [62]. The latter is used for optical transition, therefore both the total energies of the initial and final defect states are corresponding to the relaxed atomic configuration of the initial state [therefore the absorption transition level $\varepsilon_{op}(q/q^{\prime })$ is different from the emission transition level $\varepsilon_{op}(q\prime /q)$ for the same two charge states of the defect]. 

## 5. P-Type Doping

The *n*-type ZnO is easily achieved even without intentional doping. However, the *p*-type ZnO is very difficult to prepare due to the compensation effect originating from native defects or background impurities, as well as the limited solubility and inactivation of the acceptor dopants in the ZnO films. Due to the lack of reliable, reproducible and stable p-type ZnO the progress in ZnO-based devices has been limited. Intensive studies are continuously devoted to overcome this bottleneck. 

It is well known that the acceptor dopants in ZnO include group-I elements such as Li [83,84,85], Na, and K [86,87], group-Ib elements Cu [88] and Ag [89,90,91], and group-V elements such as N, P, As and Sb. Although it is theoretically suggested that group-I elements substituting for Zn possess shallow acceptor levels, it also appears that group-I elements, especially small size impurity such as Li, tend to occupy the interstitial sites (e.g., Li_i_) which act as donor defects [92]. Much effort was focused on the controllable doping of group-I elements. Lu *et al.* [84,85] deposited Li-doped ZnO films by PLD with or without using ionization source. They found that Li atoms had a larger possibility to form Li_Zn_ than Li_i,_ and this tendency was very strong under the oxygen-rich growth condition. The best result was obtained for ZnO:Li at a 0.6 at % Li content, which had the hole concentration of 6.04 × 10^17^ cm^-3^, hall mobility of 1.75 cm^2^/Vs, and resistivity of 5.9 Ωcm. Wu and Yang [86] prepared K-doped *p*-ZnO thin films using rf magnetron sputtering technique under oxygen rich atmosphere. The optimal *p*-ZnO:K films possessed a higher hole concentration of 8.92 × 10^17^ cm^-3^ and a lower resistivity of 1.8 Ωcm. Ag-doped ZnO films were fabricated by PLD [89,90] and reaction sputtering [91], using Ag_2_O as the silver dopant, and p-type ZnO could only be obtained within a narrow deposition temperature window. A neutral acceptor bound exciton emission peak of 3.317 eV was observed at 11 K [90]. Recently, several researchers have put efforts into the exploration of *p*-ZnO film by doping group-V elements as *p*-type dopants such as N [27,35,93], P [94,95,96] As [97,98,99], and Sb [100,101]. For group-V elements，Park *et al.* [92] have also given a prediction, by using first-principles pseudopotential method, that P and As are amphoteric: substitutional defects P_O_ and As_O _are deep acceptors, but due to the size mismatching to O, donor-like antisite defects P_Zn_ and As_Zn_ are more likely to form to avoid the build-up of local strains near the O site. They concluded that among these acceptors nitrogen dopant with a shallow acceptor level is a promising candidate to substitute for oxygen atoms as N_O_ in the ZnO films because of the similar ionic radius and smallest ionization energy.

To date, N-doped ZnO films have been prepared using various deposition methods such as chemical vapor deposition [27,102,103,104], spray pyrolysis [105], pulsed-laser deposition [106], implantation [107], and sputtering technology [108,109,110] using different nitrogen sources such as N_2_, NH_3_, N_2_O, Zn_3_N_2_, and MMH*_y_* (monomethyl hydrazine). However, the reliability and reproducibility in obtaining *p*-type ZnO:N is still controversial. Because of the much higher chemical activity of O compared to that of N, Zn is prone to combine with O rather than N, resulting in the N atoms being difficult to be introduced into ZnO films. To solve this problem, Yamamoto and Yoshida [111] studied the “unipolarity” doping problem in ZnO crystal, based on the results of *ab initio* electronic band structure calculations and they found that while p-type doping using the N species leads to an increase in Madelung energy n-type doping using Al, Ga or In causes a decrease in Madelung energy. Then they proposed a codoping method, which use simultaneously nitrogen acceptors and reactive III-group donors as dopants complex such as Ga–N, In–N, and Al–N, to increase the solubility of N atoms in the ZnO films and lower the acceptor level in the band gap due to strong interaction between N acceptors and reactive donor codopants. In recent years, *p*-type ZnO films were comprehensively achieved by using the codoping method [35,112,113,114,115,116,117,118,119]. Compared with Ga and In atoms, Al is more suitable as reactive donors for their superior advantages such as low cost and near containment-free material as well as the superior stability for the strong Al–N and Al–O bonds. 

**Figure 13:**
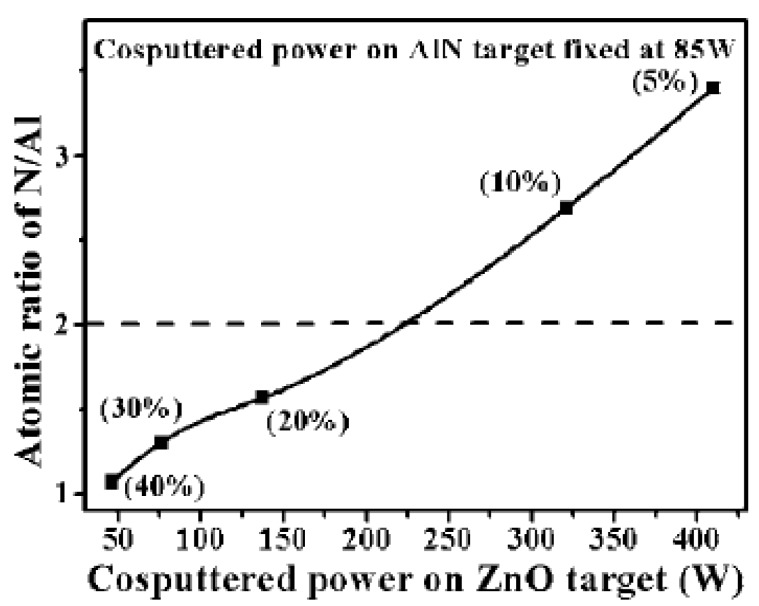
Atomic concentration of nitrogen to aluminum [N/Al] in the AlN–ZnO cosputtered films as a function of the rf cosputtering power on ZnO target (the theoretical Al atomic ratio [Al/ (Zn+Al) at %] is shown in bracket). Reprinted from Ref. [20] with permission.

In view of the formation of Al-N codoped *p*-ZnO sensitive to the deposition condition, we proposed a controllable and well-configured rf magnetron co-sputtering method to prepare Al–N codoped ZnO films by using hexagonal ZnO and AlN as targets [20]. The rf magnetron co-sputtering system used in our lab is equipped with a dual rf power supply that generate two different rf powers with synchronized phases, which has been described in Section 2. With this method the doping concentration in the deposited ZnO films could be easily controlled by the co-sputtering rf power on each target. It is found that the measured Al atomic ratio [Al/ (Zn+Al) at %] in the co-sputtered film decreases from 9.22-2.46 at. %, when the rf power supplied on the ZnO target increases from 40 to 410 W and that on AlN target is fixed at 85 W. The measured ratios are much smaller than the value theoretically evaluated from the deposition rates of the two targets, indicating the difficulty of incorporating AlN into ZnO films. The true atomic ratio of nitrogen to aluminum [N/Al in at %] in the AlN–ZnO co-sputtered films as a function of the rf co-sputtering power on the ZnO target is shown in Figure 13. The N atomic concentrations in these Al–N codoped ZnO films are higher than the Al atomic concentrations, especially for the films prepared at elevated co-sputtering powers on the ZnO target. In spite of the higher ratio [N/Al], as-deposited Al–N codoped ZnO films still show *n*-type conductive behavior, indicating the inactivity of N acceptor dopants in these Al–N codoped films deposited at room temperature. It is found that only after an adequate post-annealing treatment the N-related acceptor dopants are activated, and the resulting ZnO films exhibit *p*-type conductive behavior (Table 3).

**Figure 14:**
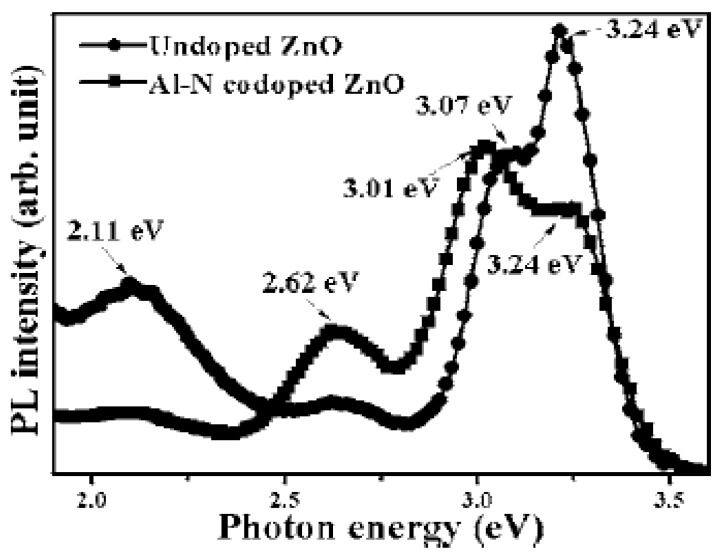
Room temperature PL spectra of the Al–N codoped [theoretical Al atomic ratio of 10%] and undoped ZnO films deposited on silicon substrates annealed at 400 °C under nitrogen ambient for 30 min. Reprinted from Ref. [20] with permission.

Annealing at 300 °C, the Al–N codoped ZnO film performed *n*-type conduction with a slightly higher electron concentration than the as-deposited film. This indicated that 300 °C was too low to activate the N-related acceptors and more donors were generated. As the annealing temperature reached 400 °C, the annealed Al–N codoped ZnO film with a hole carrier concentration of 5.04 × 10^18^ cm^−3^, mobility of 2.35 cm^2^ V^−1^s^−1^, and resistivity of 0.527 Ωcm was obtained, whereas an undoped ZnO film annealed under the same conditions showed an electron concentration of 2.57 × 10^17^ cm^−3^ and Hall mobility of 5.47 cm^2^ V^−1^s^−1^. This implied that large amounts of N-related acceptors were effectively activated and predominated over the donors in the codoped film under this annealing condition. By further increasing the annealing temperature, more donor-related defects such as *V_O_* and *V_N_* were prone to be produced, resulting in a decrease in the hole concentration. In addition, the room temperature PL spectrum also showed the features related to the activated N-related acceptors: the 60 meV redshift of the shallow level transition (from 3.07 eV in undoped ZnO film to 3.01 eV) and the suppression of oxygen-related deep level emission (2.11 eV) (Figure 14).

**Table 3:** Electrical properties of the Al–N codoped ZnO films [theoretical Al atomic ratio of 10%] deposited on silicon substrates annealed at various temperatures under nitrogen ambient for 30 min. Reprinted from Ref. [20] with permission.

Annealing temperature	Carrier concentration (cm^−3^)	Mobility (cm^2^V^−1^s^−1^)	Resistivity (Ωcm)	Carrier type
As−deposited	8.99 × 10^18^	0.96	1.97	*n*
300 °C	1.49 × 10^19^	0.83	1.38	*n*
400 °C	5.04 × 10^18^	2.35	0.527	*p*
500 °C	1.04 × 10^18^	3.64	1.65	*p*
600 °C	1.53 × 10^17^	5.03	1.34×10^1^	*p*
700 °C	5.88 × 10^15^	6.58	1.62×10^2^	*p*

In order to improve the quality of *p*-type ZnO codoped with AlN and study the function of nitrogen in these films in more detail, we [120] prepared a series of AlN codoped ZnO films using the same radio frequency (rf) magnetron cosputtering system, but under various N_2_/(N_2_ + Ar) flow ratios and with lower rf power. The rf powers of AlN and ZnO were fixed at 25 and 100 W, respectively, and the N_2_/(N_2_ + Ar) flow ratios of 0%, 4%, 8%, and 12% were used with the total flow rate kept at 50 sccm Then the prepared samples were post-annealed at 400, 450, and 500 °C for 10 min in a N_2_ ambient to activate the doping impurities. The electronic properties of samples deposited in various atmospheres and annealed at different temperatures are listed in Table 4. The highest electron concentration of samples B would be attributed to the effective substitution of Zn sites by Al atoms provided by cosputtering AlN, which also implied that at the deposition condition of B group (under pure Ar ambient) not enough N acceptors were formed. In other words, the formation of Zn–N bonds was not preferential in an insufficient N atmosphere. The sample with a lower annealing temperature of 400 °C in group C exhibited n-type conductive behavior. With the same annealing temperature, the samples in groups D and E, deposited under higher N_2_/(N_2_ + Ar) flow ratios, exhibited a high resistivity and ambiguous carrier type. However, with the intermediate post-annealing temperature at 450 °C, the deposited films of groups C, D, and E were converted from n-type into p-type conduction, indicating that the N-related acceptor dopants were activated properly by the annealing treatment at this temperature. The fact that samples D and E deposited under a higher N_2_/(N_2_ + Ar) flow ratio have a lower hole concentration was due to the self-compensation induced by the higher (N_2_)_O_ concentration within ZnO films. Moreover, the phenomenon that the carrier type of samples in C, D, and E, annealed at a higher temperature of 500 °C, changed from p-type to n-type was assumed to the dissociation of Zn–N bonds and the formation of native defects, such as oxygen vacancies. 

**Table 4:** Resistivity, Hall mobility, carrier concentration, and carrier type measured by Hall measurement for films deposited at various conditions. Reprinted from Ref. [20] with permission.

Sample group ID	Target	Gas (sccm)	Post-annealing temperature	Resistivity (Ω−cm)	Hall mobility (cm^2^/V−s)	Carrier concentration (cm^−3^)	Carrier type
A	ZnO	Ar:50	400 °C	3.67 × 10 ^−1^	3.66	4.61 × 10^18^	n
			450 °C	2.74 × 10 ^−1^	4.71	4.83 × 10^18^	n
			500 °C	2.09 × 10 ^−1^	4.79	6.57 × 10^18^	n
B	ZnO	Ar:50	400 °C	1.45 × 10 ^−1^	4.02	1.07 × 10^19^	n
	AlN		450 °C	2.92 × 10 ^−2^	4.42	4.85 × 10^19^	n
			500 °C	2.73 × 10 ^−2^	4.64	4.95 × 10^19^	n
C	ZnO	Ar:48	400 °C	7.44	1.56	7.23 × 10^17^	n
	AlN	N_2_:2	450 °C	3.88	1.35	1.17 × 10^18^	p
			500 °C	1.67 × 10 ^−1^	4.42	8.31 × 10^18^	n
D	ZnO	Ar:46	400 °C	1.39 × 10 ^4^	3.51	1.28 × 10^14^	p/n
	AlN	N_2_:4	450 °C	6.12 × 10 ^2^	1.24	8.21 × 10^15^	p
			500 °C	1.53 × 10 ^1^	4.13	9.92 × 10^15^	n
E	ZnO	Ar:44	400 °C	1.28 × 10 ^4^	2.12	2.28 × 10^14^	p/n
	AlN	N_2_:6	450 °C	1.13 × 10 ^3^	3.14	1.76 × 10^15^	p
			500 °C	2.81 × 10 ^2^	3.25	6.95 × 10^15^	n

**Figure 15:**
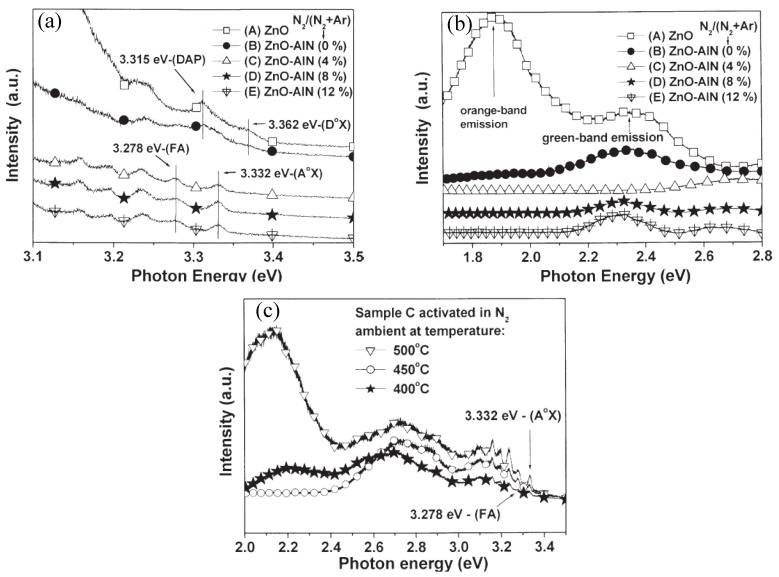
LTPL spectra (10 K) of ZnO and AlN–ZnO [N_2_/(N_2_ + Ar) = 0%, 4%, 8%, 12%] films annealed at 450 °C (a) in UV emission range. (b) in deep level emission range. (c) LTPL spectra of the AlN–ZnO [N_2 _/(N_2_+Ar) = 4%] films annealed at 400, 450, and 500 °C, respectively. Reprinted from Ref. [120] with permission.

All these suppositions were confirmed by the results of XRD, low temperature PL spectra (LTPL) and time-resolved PL spectra (TRPL) measurements. Figure 15(a) shows the LTPL spectra of undoped and AlN codoped ZnO films post-annealed at 450 °C in UV range. The emission peak at 3.362 and 3.315 eV of sample A and B were assigned as the neutral donor bound exciton (D°X) and donor-acceptor pair (DAP) transition, respectively. A new strong peak at 3.332 eV clearly observed for samples C, D, and E was attributed to the neutral acceptor bound exciton (A°X), while the peak at 3.278 eV was labeled as the recombination emissions of free electron to acceptor hole level (FA) due to the nitrogen in the oxygen site N_O_. LTPL in deep level emission range of ZnO, AlN–ZnO [N_2_/(N_2_ + Ar) = 0%, 4%, 8%, 12%] films annealed at 450 °C are shown in Figure 15(b). Two emission bands at 2.35 and 1.89 eV, corresponding presumably to oxygen vacancies and oxygen interstitials, respectively, in sample B were weaker than that in sample A, implying less oxygen defects existed in sample B. This fact suggested that the higher electron concentration of sample B (listed in Table 4) might be attributed to the substitutional Al atoms on Zn sites instead of oxygen vacancies. 

Compared to the case of the sample C, more N_2_ on O substitution sites (*N*_2_)*_O_* simultaneously existed in samples D and E, which resulted in the larger lattice constants as identified by the XRD experimental results (not shown here). This was consistent with the fact that samples D and E were deposited under higher N_2_/(N_2_ + Ar) flow ratios. Furthermore, the fact that the intensity of 2.35 eV emission band in samples D and E is higher than that in sample C implies more O vacancies existed in samples D and E, which can be tentatively interpreted as the result of compensation for the existence of more (*N*_2_)*_O_* in the film. Because both O vacancies and (*N*_2_)*_O_* serve as donors in ZnO film, N acceptors formed in samples D and E were partly compensated by them and resulted in a lower hole concentration as seen in Table 4. Figure 15(c) is the LTPL spectra of sample C ([N_2_ /(N_2_+Ar) = 4%]) annealed at 400, 450, and 500 °C, respectively. The broad blue-green emission band around 2.4–2.8 eV was originated from the deep level caused by the dopant-induced defects. The other broad deep level emission at 2.15 eV was regarded as the oxygen-related emission. As shown in Figure 15(c), the emission induced by the dopant increased and the oxygen-related emission decreased, when the annealing temperature increased from 400 to 450 °C for sample C. This implies that more nitrogen atoms occupied the oxygen vacancy sites to form N_O_ acceptors, which interprets the stable p-type behavior for the sample in group C annealed at 450 °C. The degradation of the *p*-type behavior for samples annealed at higher temperatures was attributed to the dissociation of Zn–N bonds and Zn–O bonds. 

The results discussed above lead us to the conclusion that high quality *p*-type ZnO films can be obtained by co-sputtering of ZnO and AlN under an adequate N_2_/(N_2_ + Ar) flow ratio of 4% and post-annealing at 450 °C.

## 6. Nanostructured ZnO Materials

It is well known that nanometer semiconductor may have superior optical and electrical properties than bulk crystals owing to quantum confinement effects. Great efforts have been devoted to the nano-science and technology in the world. Nanostructured ZnO is also a hot topic in the research community, especially one-dimentional (1D) ZnO nanostructures. Large amount of research reports on 1D nanostructured ZnO of different shapes have been published and comprehensively reviewed [121]. Here we will not review all the aspects of the attracting field but just focus on limited topics. Many methods have been used to obtain ZnO Nanowires or nanorods. For example, Lee *et al.* [122] have used MOCVD to grow well-aligned, single-crystalline ZnO nanowires on GaAs substrates. Yuan *et al.* [123] reported that well-aligned ZnO nanowire (NW) arrays with durable and reproducible p-type conductivity were synthesized on sapphire substrates via vapor-liquid-solid growth by using N_2_O as a dopant source. Low-temperature growth routes for ZnO nanorods have also been reported [124,125]. 2D ZnO nanostructures have large surface area and thus are suitable for potential applications in nanoscale optoelectronics, sensors, dye-sensitized solar cells, and light emitters *etc*.

In our lab, several deposition methods have been used to grow nanostructured ZnO layers, such as low pressure vapor phase transport process [126], carbothermal reduction, deposition using vapor cooling condensation through porous Al template, and hydrothermal method. 

**Figure 16:**
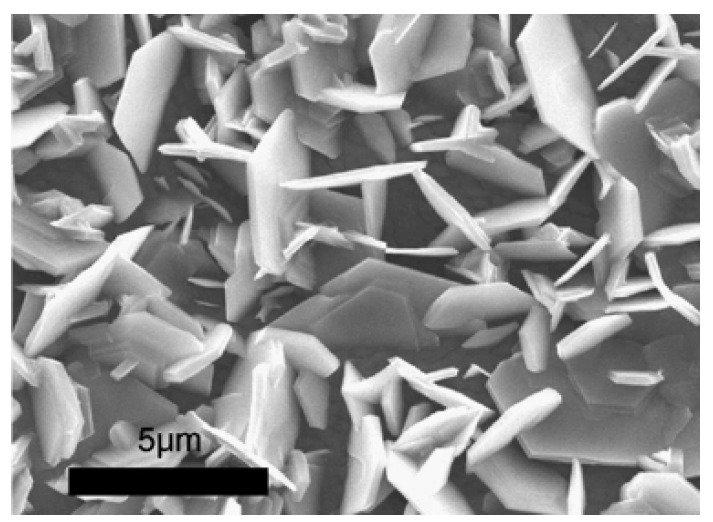
SEM images of ZnO nanodisks grown at 1000 °C in flowing Ar (99 sccm)/O_2_ (1 sccm) and then subjected to furnace cooling. Reprinted from Ref. [59] with permission.

We fabricated In-doped ZnO nanodisks (Figure 16) by carbothermal reduction [59], and found that the In doping seems to increase the activation energy required for the growth unit joining with the (0002) surface, resulting in the suppression of the growth along the [0001] direction. But the air-cooled (cooled in air after growth by taking the samples off the furnace) ZnO nanodisks showed a very strong green emission, which can be attributed to the defects produced by In doping. However, furnace cooling (naturally cooled to room temperature in the furnace) in conjunction with introducing O_2_, around 1.0%, into flowing Ar during growth significantly enhanced the growth rate and UV emission of ZnO nanodisks, while the green emission was significantly suppressed when the oxygen concentration was increased from 0.5 to 1%. The latter can be attributed to the reduction of oxygen vacancies and surface defects in ZnO nanodisks. It is noticed that when the oxygen concentration was increased to 10%, the intensity of UV emission was considerably reduced. It is conceived that at a higher oxygen partial pressure many Zn vacancies may form in the ZnO nanodisks, resulting in suppression of UV emission.

ZnO nanorod arrays with different densities and sizes were also fabricated by using a chemical vapor transport process in our lab [31]. The source material, Zn powder (purity 99.5%), was placed at the sealed end of quartz tube in a furnace. The system was maintained at a pressure of about 30 Torr. Argon gas was used as the carrier gas with a constant flow rate of 200 sccm. Oxygen gas with 30 sccm flow rate was introduced into the furnace at 450 °C. Samples A, B, C, and D were placed at ~13, 18, 23, and 28 cm from the center of furnace with their temperatures being ~640, 630, 560, and 470 °C, respectively. The Zn source was maintained at 650 °C for 30 min. Then the furnace was cooled down to room temperature naturally. 

**Figure 17:**
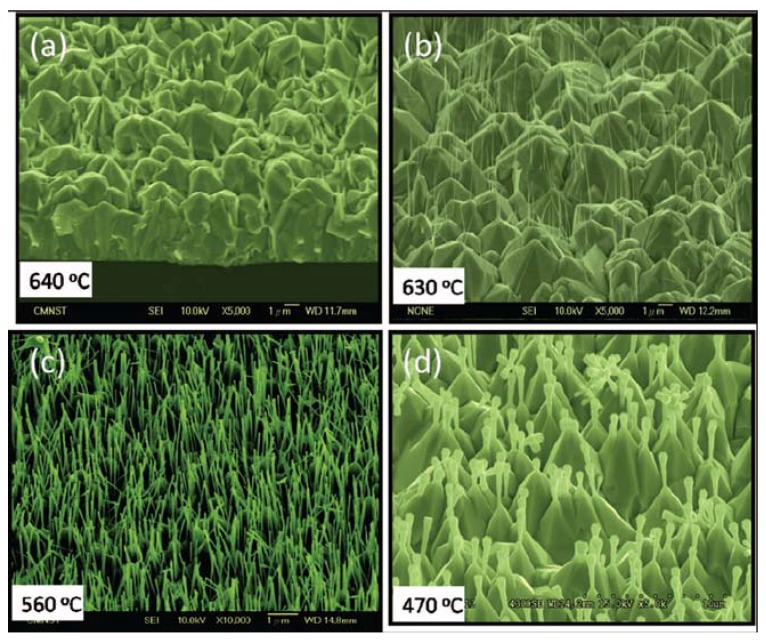
SEM images of samples A, B, C, and D grown at (a) 640, (b) 630, (c) 560 and (d) 470 °C, respectively. The magnifications are 5000 for a, b, and d and 10000 for c. Reprinted from Ref. [31] with permission.

Figure 17 shows SEM images of the samples A, B, C, and D. Sample A consists of micropyramids on the Si substrate. Short nanorods formed only on the tips of some of micropyramids. Sample B consists of micropyramids with thin nanorods on their tips. Sample C shows high-density ZnO nanorod arrays on the tips of small micropyramids. However, the morphology of sample D differs from the other samples. The tip size of the nanorods grown on the pyramids is larger than the root size. The growth mechanism of nanorod array was analyzed. In brief, the population of ZnO nanorods was controlled by the secondary growth of nanorods on the tips of primarily grown pyramids. The hexagonal prisms and consequent pyramids are attributed to the difference in the growth rate of different crystal planes. The overgrowth of ZnO nanorods on the micropyramidal tips is due to the orientation adhesion. The density and size of these nanorods are influenced by the difference of saturation pressure at different temperature and the transport of source material by the carrying gas. The dense nanorods with small size exhibited excellent field emission properties. 

We also fabricated vertically well-aligned ZnO nanowires by spin-on-glass technology on ZnO:Ga/glass templates [127], which has been used for constructing photodetectors.

In addition to the preparation of the nanostrutured ZnO, their optical properties are another important issue to be exploited. Generally the optical properties of nanostructured semiconductors depend strongly on their size and shape [128], which is a crucial issue in nanophysics due to their role in carrier relaxation [129] and very important for many applications such as single photon source [130] and high frequency acoustic emitter etc [131]. For example, Fonoberov *et al.* studied theoretically the optical properties of zero-dimensional ZnO nanostructures or quantum dots (QDs). They found that depending on the fabrication technique and ZnO QD surface quality, the origin of UV photoluminescence in ZnO QDs with diameters from 2–6 nm was due to the recombination of either confined excitons or surface-bound ionized acceptor–exciton complexes. In the latter case the Stokes shift of about 100–200 meV was observed in the photoluminescence spectrum. They also found that the radiative lifetime of the confined exciton and that of the ionized donor–exciton complex were almost the same. The lifetimes in both cases decreased with QD size and were about an order of magnitude less (for QD of radius *R*~2 nm) than the bulk exciton lifetime. For the QD with diameter 5 nm the lifetime of 38 ps was obtained, in agreement with the conclusion of Bahnemann *et al.* [132]. This is beneficial for optoelectronic device applications. On the other hand, the radiative lifetime of the ionized acceptor–exciton complex increased with QD size very fast, particularly for QD of *R*~2 nm, the lifetime was about two orders of magnitude larger than the bulk exciton lifetime. Thereby they proposed to use the exciton radiative lifetime as a probe of the exciton localization. Laser action from nanostructured ZnO is another interesting topic that has been studied widely. As an example, X.H. Han *et al.* [133] demonstrated the lasing from high density well-aligned ZnO nanorod arrays synthesized by an improved vapor transport method on ZnO thin films.

**Figure 18:**
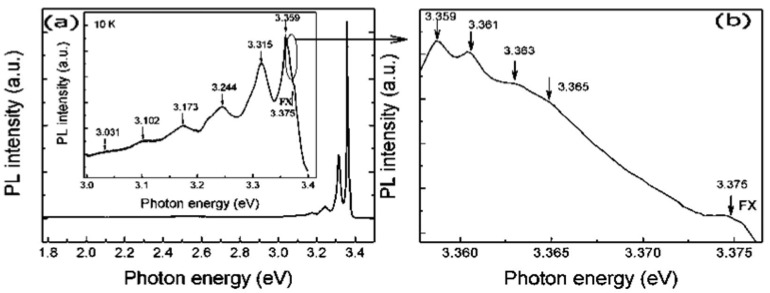
PL spectrum of ZnO nanowires measured at 10 K, (a) for photon energy ranging from 1.77–3.5 eV. The inset is the part in the near-band edge region (3.0–3.4 eV) in a magnified scale. (b) High resolution high energy shoulder in near-band edge emission. Reprinted from Ref. [126] with permission.

Short wavelength luminescence of nanostructured ZnO is of great interest due to its potential optoelectronic application. Because of its fundamental importance we studied exciton related interaction in the ZnO nanostructure. We successfully fabricated high quality ZnO nanowires with a diameter of about 70 nm and 1000 nm in length on Si (100) substrates by a low pressure vapor phase transport process [127], and then studied the free excitonic transition in individual ZnO nanowires. Figure 18(a) shows the PL spectrum of ZnO nanowires measured at 10 K, in which strong near-band-edge (NBE) emission peaks are observed, and deep level visible emissions relating to structural defects are almost negligible. This fact suggests a low defect density for the as-grown nanowires. The clear observation of the FX peak (at 3.375 eV), the neutral-donor bound exciton (*D*^0^*X at* 3.359 eV), the 1-LO(71 meV), 2-LO, 3-LO, and 4-LO phonon replicas of donor acceptor pair (DAP at 3.315 eV ) transition, and the peaks at high energy shoulder (attributed to bound excitons at different unintentional impurities) further indicates (besides the FE-SEM image and the XRD pattern ) that ZnO nanowires are of high optical quality.

## 7. ZnO Based Light Emitting Diodes

Due to the progress in preparation of ZnO materials, especially *p*-ZnO materials, more research efforts have been devoted to the exploration of various ZnO-based short wavelength light-emitting diodes. The progress in the aspect goes ahead continuously and new improvements have been demonstrated in recent years, as summarized in a recent publication [134]. 

Firstly, the exploration of ZnO *p-n* homojunction LEDs is still an attractive research topic. The progress achieved in this direction is closely related to the improvement in preparation of *p*-type ZnO materials. It is not difficult to list a few newly reported examples of ZnO *p*-*n* junction LEDs, where *p*-ZnO was obtained by using different dopants, such as NO [135], N_2_O [136], N_2_-O_2_ [137], N-Al [138], N-In [139], and Sb [101]. Various deposition methods, including plasma-assisted molecular beam epitaxy, dc reactive magnetron sputtering, MOCVD, ultrasonic spray pyrolysis and so on were used to deposit the ZnO layers. In most cases, the resulting ZnO *p-n* junction LEDs emitted blue-violet electroluminescence under forward bias at low temperature, which was attributed to donor-acceptor pair recombination in *p*-ZnO. Some works also reported the emission from deep defect levels. It is noted that device performance improved, but the EL was still quenched substantially at room temperature. This can be ascribed to the imperfection of the *p*-ZnO. 

In view of the fact that the high quality *p*-ZnO is still not available, many efforts have continuously focused on an alternative type of device structures, the *n*-ZnO based heterojunction (e.g., [140]), in which the active *n*-ZnO layer is deposited on other *p*-type materials to form *n*-ZnO based *p*-*n* junction LEDs. 

Rogers *et al.* [141] fabricated a *n*-ZnO/ *p*-GaN:Mg heterojunction LED on *c*-Al_2_O_3_ substrates using pulsed laser deposition for the ZnO and metal organic chemical vapor deposition for the GaN:Mg. Room temperature electroluminescence of the LED showed an emission peaked at 375 nm, the same as the photoluminescence of the ZnO layer, indicative of a near band edge excitonic emission. Over the injection current range from 500–875 mA the light-current (*L*-*I*) characteristic of spectral intensity versus current appears fairly linear.

Long *et al.* [142] reported that ultraviolet light-emitting diodes based on ZnO/NiO heterojunctions were fabricated on commercially available *n*^+^-GaN/sapphire substrates using a radio frequency magnetron sputtering system. Near band edge emission of ZnO peaking at 370 nm was achieved at room temperature when the devices were under sufficient forward bias. It is also demonstrated that the device performance was improved by inserting an electron blocking *i*-Mg_1−*x*_Zn*_x_*O (0 < *x* < 1) layer.

Titkov, Delimova *et al.* [143] demonstrated white electroluminescence (EL) from ZnO/GaN structures fabricated by pulsed laser deposition of ZnO:In onto MOCVD-grown GaN:Mg/GaN structures on Al_2_O_3_ substrates. The white EL was produced by superposition of two strongest emission lines, a narrow blue line at 440 nm and a broad yellow band around 550 nm, which was attributed to the high density of structural defects at the n-ZnO/p-GaN interface.

Bayram *et al.* [144] reported a hybrid green light-emitting diode comprised of *n*-ZnO/ (InGaN/GaN) multi-quantum-wells/*p*-GaN, which was grown on semi-insulating AlN/sapphire using pulsed laser deposition for the *n*-ZnO and metal organic chemical vapor deposition for the other layers. LEDs showed a turn-on voltage of 2.5 V and a room temperature EL centered at 510 nm.

Besides the development of various device structures, the surface passivation was also used to improve the performance of the devices. For example, Yu-Lin Wang *et al.* [145] investigated the passivation effects of PECVD-deposited SiO_2_ and SiN_x_ on ZnO-based heterojunction *p*-*i*-*n* LEDs.

In recent years, *p*-ZnO has also been used to construct hetrostructure LEDs in combination with other materials. However, many of the devices yield deep-level related emissions with none or small UV emissions. Mandalapu *et al.* [146] reported the Sb-doped *p*-ZnO/*n*-Si heterojunction diode fabricated by MBE. Near-band edge and deep-level emissions were observed from the LED devices at both low temperatures and room temperature, which is due to band-to-band and band-to-deep level radiative recombination in ZnO, respectively.

Different devices of complex structure have been reported. Kim *et al.* [147] fabricated ZnO-based LEDs using ZnO:P/Zn_0.9_Mg_0.1_O/ZnO/Zn_0.9_Mg_0.1_O/ZnO:Ga *p*-*i*-*n* heterostructures, which showed EL coming from deep level emission at low forward bias and near band edge ultraviolet emission at high bias. Ryu *et al.* [45] fabricated ZnO-based ultraviolet LEDs comprised of a ZnO/BeZnO MQW active layer between *n*-type and *p*-type ZnO and Be_0.3_Zn_0.7_O layers. Arsenic and gallium were used as dopants for *p*-type and *n*-type layers. The EL emission was attributed to the active layer. This kind of device structure was also used for laser diode [148]. The lasing mechanism is inelastic exciton-exciton collision. Chu *et al.* [149] also reported ZnO based quantum well diode lasers, where Sb-doped *p*-type ZnO/Ga-doped *n*-type ZnO with an MgZnO/ZnO/MgZnO quantum well embedded in the junction was grown on Si by molecular beam epitaxy. The diodes emit lasing at room temperature with a very low threshold injection current density of 10 A/cm^2^. The lasing mechanism is exciton-related recombination. 

In addition, ZnO based LEDs and laser diodes of MIS or MOS structure were also demonstrated, for example, a MIS structure LED [150], and the electrically pumped UV random lasing in the *c*-axis oriented ZnO-based MOS (Au/SiO*_x_*(*x* < 2) /ZnO) device [151]. 

**Figure 19:**
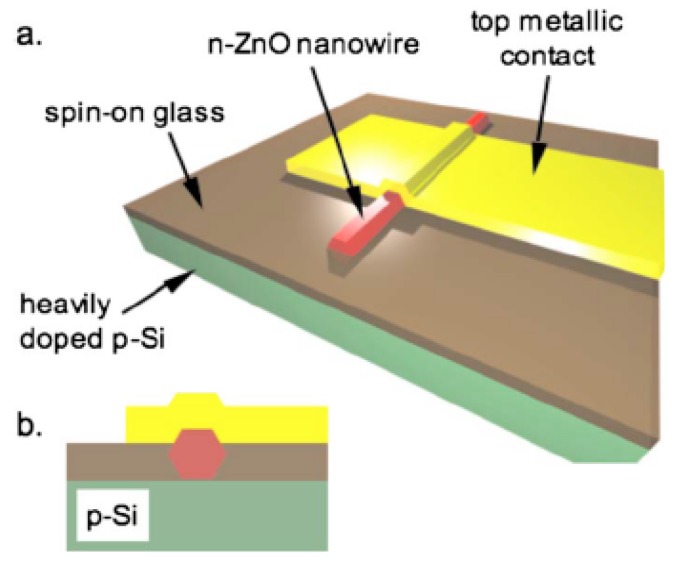
Schematics of the *n*-ZnO/ *p*-Si nanowire LEDs. a. Top view, b. cross-sectional view. Reprinted from Ref. [156] with permission.

Finally, it should be of interest to mention the progress in nanostructured ZnO LEDs. Yang *et al.* [152] demonstrated efficient UV and red EL at room temperature from ZnO nanorod array light-emitting diodes, where the *p*-type ZnO was formed by As^+^ ion implantation into the as-grown ZnO nanorods. Jeong *et al.* [153] reported ZnO nanowire-array-embedded *n*-ZnO/*p*-GaN heterojunction light-emitting diodes, showing electroluminescence emission of the wavelength of 386 nm come out from ZnO nanowire. Bao *et al.* [154] constructed a single nanowire light-emitting diode. A hybrid light-emitting *p*-*n* junction diode has been produced using ZnO nanorods as the *n*-type material [155]. Zimmler *et al.* [156] recently fabricated ZnO nanowire LEDs on a heavily doped *p*-type silicon substrate (Figure 19). The electroluminescence of the single wire LED was attributed to bound- and free-exciton related recombination, together with their LO phonon replicas.

The random lasing action was also demonstrated by Leong and Yu [157] in *p*-SiC(4H)/*i*-ZnO-SiO_2_ nanocomposite/*n*-ZnO:Al heterojunction (*p-i-n*) laser diodes. The intrinsic layer, inserted between the *n-* and *p*-injection layers, consisted of ZnO powder embedded in a SiO_2_ matrix, prepared by the sol–gel technique. The lasing was due to the ZnO clusters.

**Figure 20:**
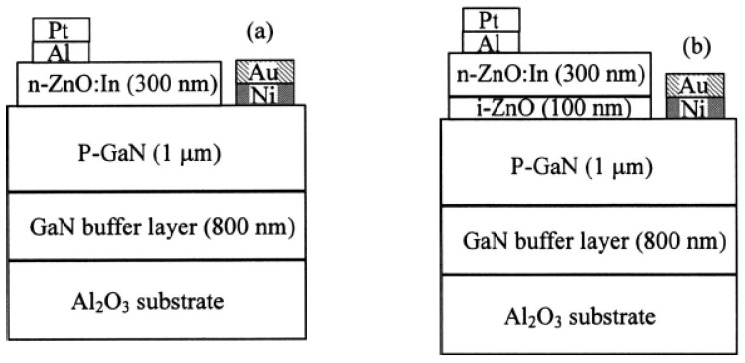
Schematic of hybrid (a). *p*-*n* and (b). *p*-*i*-*n* heterojunction LEDs. Reprinted from Ref. [39] with permission.

It is noted from the above discussion that defects in ZnO, including undoped, manifest itself frequently in the luminescence. In order to fabricate ZnO based UV LED, ZnO of very few deep level defects has to be used for device fabrication. We have discussed above that the vapor cooling condensation method can deposit ZnO layer with much less oxygen vacancies. Using the method to deposit both doped and undoped ZnO layers, we have developed two kinds of *n*-ZnO based hybrid LEDs: the *p*-GaN/*n*-ZnO:In (*p*-*n*) and *p*-GaN/*i*-ZnO/*n*-ZnO:In (*p*-*i*-*n*) heterojunction light-emitting diodes [39]. The structures of both types of diodes are shown schematically in Figure 20.

**Figure 21:**
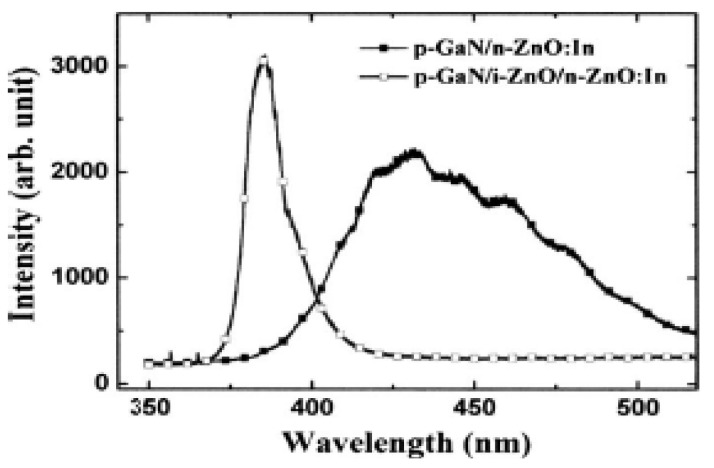
The room-temperature EL spectra of the *p*-*n* and *p*-*i*-*n*. Reprinted from Ref. [39] with permission.

The room temperature current-voltage characteristics clearly demonstrated a rectifying diodelike behavior for both devices. The EL spectra of the fabricated p-n and p-i-n devices under forward bias are shown in Figure 21 The EL spectrum of *p*-GaN/*n*-ZnO:In heterojunction LED consists of a broad emission band at 432 nm, which is attributed to the transition from the conduction band to the acceptor level in the Mg doped *p*-GaN when electrons are injected from the *n*-ZnO:In into the Mg-doped *p*-GaN. It is interesting that the EL spectrum of *p-i-n* device showed a pure UV band at 385 nm, which is consistent with the PL spectrum of the ZnO layer deposited with the vapor cooling condensation method. It indicates that the EL emission of the *p-i-n* LEDs comes from ZnO layer of the heterostructure, the undoped *i*-ZnO layer inserted between *n*-ZnO:In and *p*-GaN. It is due to the fact that the holes from *p*-GaN and electrons from *n*-ZnO:In are injected into the *i*-ZnO layer and radiatively recombined there. It is clear that the UV EL of thus developed *p*-*i*-*n* heterostructure is closely related to the fact that the inserted *i*-ZnO layer, deposited by the vapor cooling condensation method, has much less luminescent defects. It is also worth while to point out that the developed *p-i-n* LED is of potential applications in UV LEDs for high-temperature operation. [39]

**Figure 22:**
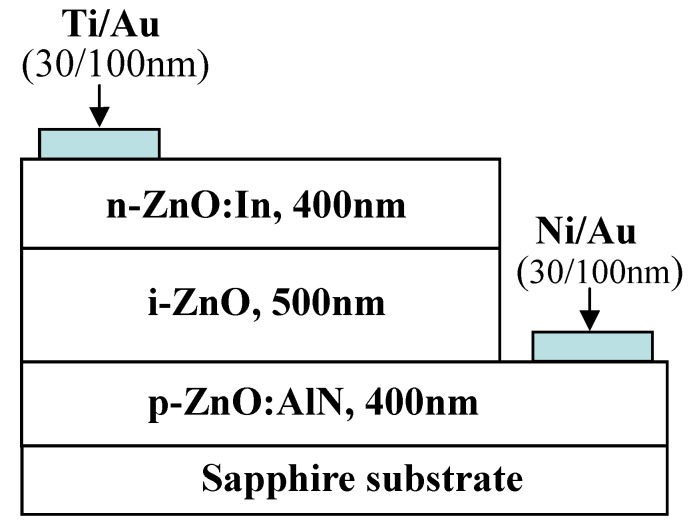
Schematic of the light-emitting diodes *p*-ZnO:AlN/*i*-ZnO/*n*-ZnO:In. Reprinted from Ref. [158] with permission.

**Figure 23:**
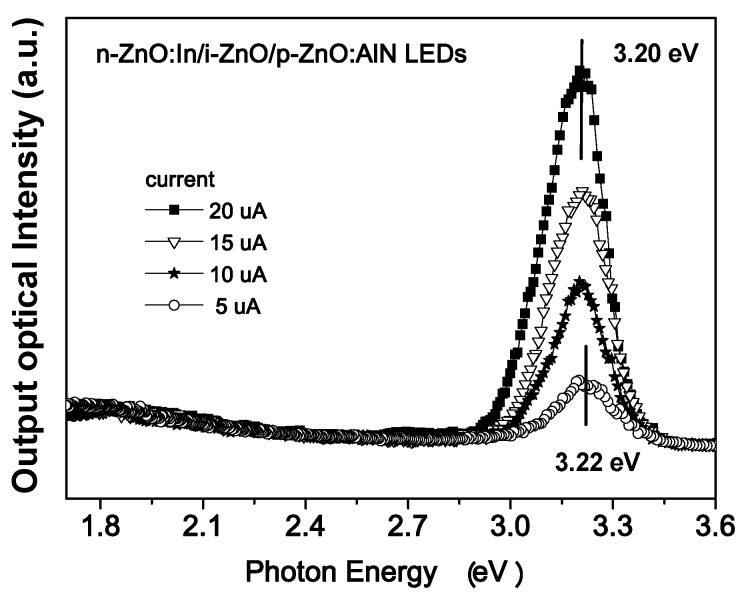
Room-temperature electroluminescence (EL) spectra of the *p-i-n* ZnO-based LEDs under different operation currents. Reprinted from Ref. [158] with permission.

In a recent investigation, we succeeded in fabricating purely ZnO based *p-i-n* UV LEDs [158]. Figure 22 shows the schematic diagram of the fabricated *p*-ZnO:AlN/*i*-ZnO/n-ZnO:In LED. The AlN codoped p-ZnO layer was deposited on sapphire substrates by using the radio frequency (RF) magnetron co-sputtering system with a dual RF power supply. The *i*-ZnO and *n*-type ZnO:In films were fabricated by the vapor cooling condensation method. The resultant LEDs showed a UV EL peaked at ~3.2 eV, operated under different injection currents at room temperature as shown in Figure 23. The observed EL band was also attributed to the radiative recombination in the *i*-ZnO layer where the deep-level defects were very few as demonstrated previously. 

**Figure 24:**
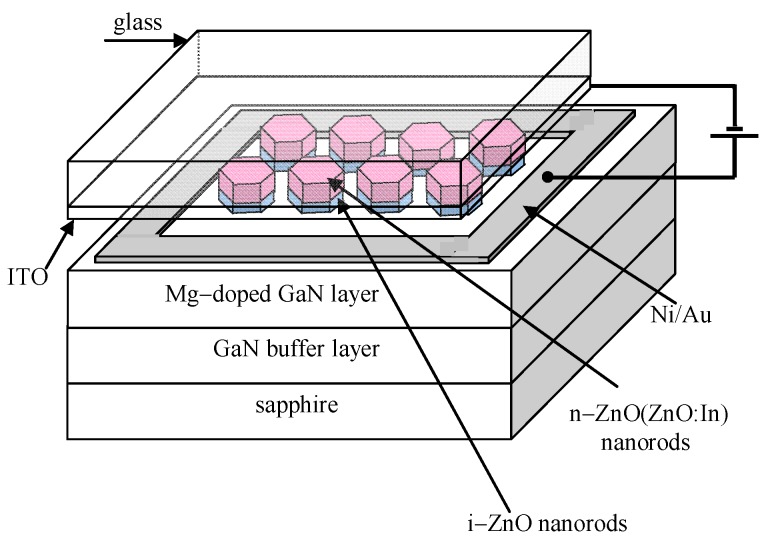
Schematic of *p*-GaN layer/*i*-ZnO nanorod array/*n*-ZnO:In nanorod array heterostructured LEDs.

We also succeeded in fabricating ZnO nanorod-based heterostructured ultraviolet LEDs [159]. Figure 24 shows the schematic of the built *p*-GaN layer/*i*-ZnO nanorod array/*n*-ZnO:In nanorod array heterostructured LEDs. Both *i*-ZnO and *n*-ZnO:In nanorod arrays in the structure were grown on a *p*-GaN layer through an anodic alumina membrane (AAM) template using the vapor cooling condensation method. 

**Figure 25:**
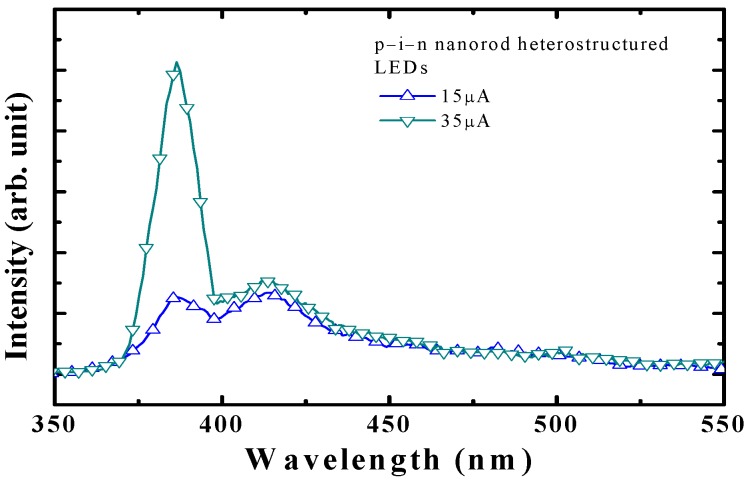
Room temperature EL spectra of the ZnO nanorod based heterostructured *p-i-n* light-emitting diodes.

Figure 25 shows the room temperature EL spectra of the ZnO nanorod based *p-i-n* heterostructured LEDs operated at 15 μA and 35 μA. In particular, the *p-i-n* devices exhibit a UV EL emission centering at 386 nm, which becomes dominant emission at higher injection current of 35 μA. This UV band is attributed to the near-band-edge emission of the *i*-ZnO nanorod array of the *p-i-n* structure.

**Figure 26:**
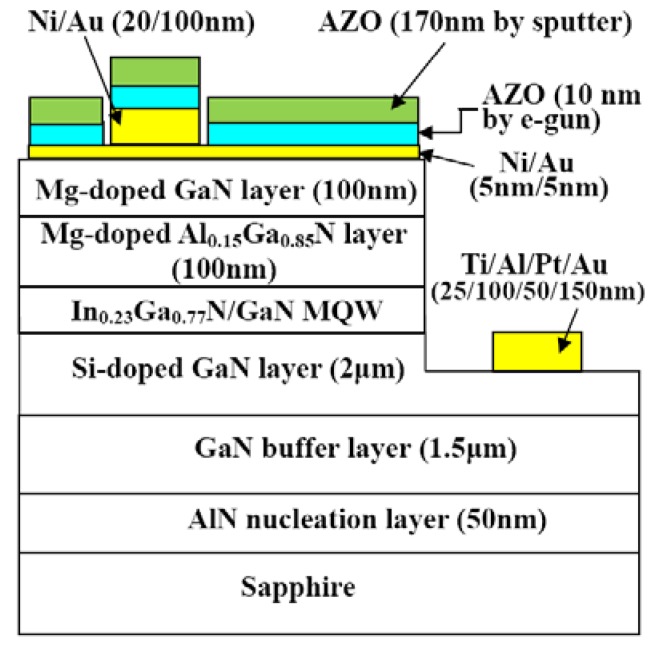
Structures of LEDs with the periphery of bonding pad disconnected with the AZO film. Reprinted from Ref. [160] with permission.

Nanostructure can also been used to improve the light extraction efficiency. In a recent work of our laboratory [160], Al-doped ZnO (AZO) films with Al nano clusters embedded were deposited by magnetron co-sputtering system, serving as the transparent conductive layer (to replace ITO film) of GaN-based light-emitting diodes, as shown schematically in Figure 26. 

**Figure 27:**
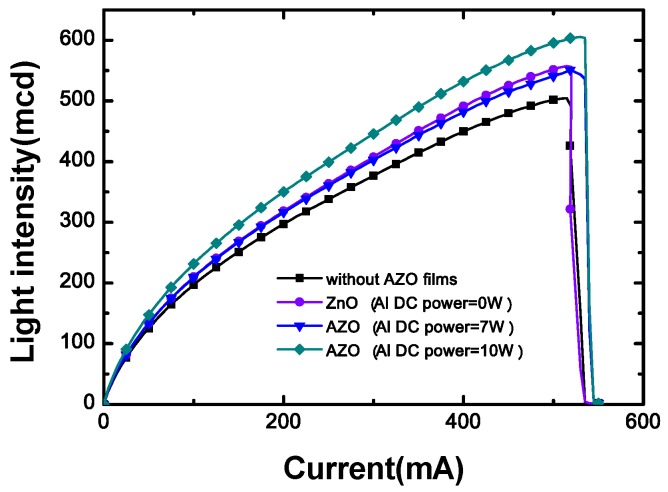
Light output power-current characteristics of LEDs with and without AZO films deposited under various Al dc power. Reprinted from Ref. [160] with permission.

The fabricated LEDs with AZO film prepared at a higher DC Al co-sputtering power (10 W) show an increase of 20% in the light output power compared to the conventional LEDs, which is 10% more than that of LEDs with AZO prepared at Al co-sputtering power of 7 W. The extra increase in the light output power was attributed to the formation of Al nanoclusters in AZO under higher Al sputtering power, which scattered the emitted light outward and hence increased the light extraction (Figure 27). 

## 8. Summary

ZnO related materials have been attracting intensive investigation due to inherent advantages and potential applications. In this paper, the recent progress on some aspects of the active research field, related to applications for the light-emitting diodes, has been reviewed in combination with the works carried out in our lab. One can feel that great progress has continuously been achieved, but there is still a long way to go for realizing extensive practical applications. Further efforts should be continued in various aspects of investigation for reaching the final goal. 

## References

[1] Özgür Ü., Alivov Ya. I., Liu C., Teke A., Reshchikov M.A., Doğan S., Avrutin V., Cho S.-J., Morkoçd H. (2005). A comprehensive review of ZnO materials and devices. J. Appl. Phys..

[2] Klingshirn C. (2007). ZnO: From basics towards applications. Phys. Stat. Sol. B.

[3] Ohshima E., Ogino H., Niikura I., Maeda K., Sato M., Ito M., Fukuda T. (2004). Growth of the 2-in-size bulk ZnO single crystals by the hydrothermal method. J. Cryst. Growth.

[4] Sekiguchi T., Miyashita S., Obara K., Shishido T., Sakagami N. (2000). Hydrothermal growth of ZnO single crystals and their optical characterization. J. Cryst. Growth.

[5] Sakagami N., Yamashita M., Sekiguchi T., Miyashita S., Obara K., Shishido T. (2001). Variation of electrical properties on growth sectors of ZnO single crystals. J. Cryst. Growth.

[6] Li W.J., Shi E.W., Zhong W.Z., Yin Z.W. (1999). Growth mechanism and growth habit of oxide crystals. J. Cryst. Growth.

[7] Shiloh M., Gutman J. (1971). Growth of ZnO single crystals by chemical vapour transport. J. Cryst. Growth.

[8] Matsumoto K., Noda K. (1990). Crystal growth of ZnO by chemical transport using HgCl2 as a transport agent. J. Cryst. Growth.

[9] Look D.C., Reynolds D.C., Sizelove J.R., Jones R.L., Litton C.W., Cantwell G., Harsch W.C. (1998). Electrical properties of bulk ZnO. Solid State Commun..

[10] Nause J., Nemeth B. (2005). Pressurized melt growth of ZnO boules. Semicond. Sci. Technol..

[11] Schulz D., Ganschow S., Klimm D., Neubert M., Rossberg M., Schmidbauer M., Fornari R. (2006). Bridgman-grown zinc oxide single crystals. J. Crystal Growth.

[12] Lee C.T., Su Y.K., Wang H.M. (1987). Effect of r.f. sputtering parameters on ZnO films deposited onto GaAs substrates. Thin Solid Films.

[13] Lee C.T., Su Y.K., Chen S.L. (1989). Dependence of ZnO films on sputtering parameters and SAW device on ZnO/InP. J. Cryst. Growth.

[14] Gardeniers J.G.E., Rittersma Z.M., Burger G.J. (1998). Preferred orientation and piezoelectricity in sputtered ZnO films. J. Appl. Phys..

[15] Jeong S., Kim B., Lee B. (2003). Photoluminescence dependence of ZnO films grown on Si(100) by radio-frequency magnetron sputtering on the growth ambient. Appl. Phys. Lett..

[16] Kim K.K., Song J.H., Jung H.J., Choi W.K., Park S.J., Song J.H. (2000). The grain size effects on the photoluminescence of ZnO/α-Al_2_O_3_ grown by radio-frequency magnetron sputtering. J. Appl. Phys..

[17] Chen P.L., Ma X.Y., Yang D.R. (2007). Ultraviolet electroluminescence from ZnO/p-Si heterojunctions. J. Appl. Phys..

[18] Liu D.S., Tsai F.C., Lee C.T., Sheu C.W. (2008). Properties of zinc oxide films cosputtered with aluminum at room temperature. Jpn. J. Appl. Phys..

[19] Lai L.W., Lee C.T. (2008). Investigation of optical and electrical properties of ZnO thin films. Mater. Chem. Phys..

[20] Liu D.S., Sheu C.S., Lee C.T. (2007). Aluminum-nitride codoped zinc oxide films prepared using a radio-frequency magnetron cosputtering system. J. Appl. Phys..

[21] Vispute R.D., Talyansky V., Choopun S., Sharma R.P., Venkatesan T., He M., Tang X., Halpern J.B., Spencer M.G., Li Y.X., Salamanca-Riba L.G., Iliadis A.A., Jones K.A. (1998). Heteroepitaxy of ZnO on GaN and its implications for fabrication of hybrid optoelectronic devices. Appl. Phys. Lett..

[22] Bhosle V., Prater J.T., Yang F., Burk D., Forrest S.R., Narayan J. (2007). Gallium-doped zinc oxide films as transparent electrodes for organic solar cell applications. J. Appl. Phys..

[23] Fons P., Iwata K., Niki S., Yamada A., Matsubara K. (1999). Growth of high-quality epitaxial ZnO films on α-Al_2_O_3_. J. Cryst. Growth.

[24] Chen Y., Bagnall D.M., Koh H.J., Park KT., Hiraga K., Zhu Z.Q., Yao T. (1998). Plasma assisted molecular beam epitaxy of ZnO on c -plane sapphire: Growth and characterization. J. Appl. Phys..

[25] Mamdalapu L.J., Xiu F.X., Yang Z., Liu J.L. (2007). Al/Ti contacts to Sb-doped p-type ZnO. J. Appl. Phys..

[26] Zhu H., Shan C.X., Li B.H., Zhang J.Y., Yao B., Zhang Z.Z., Zhao D.X., Shen D.Z., Fan X.W. (2009). Ultraviolet electroluminescence from MgZnO-based heterojunction light-emitting diodes. J. Phys. Chem. C.

[27] Sun J.C., Liang H.W., Zhao J.Z., Bian J.M., Feng Q.J., Hu L.Z., Zhang H.Q., Liang X.P., Luo Y.M., Du G.T. (2008). Ultraviolet electroluminescence from n-ZnO:Ga/p-ZnO:N homojunction device on sapphire substrate with p-type ZnO:N layer formed by annealing in N_2_O plasma ambient. Chem. Phys. Lett..

[28] Liu Y., Gorla C.R., Liang S., Emanetoglu N., Lu Y., Shen H., Wraback M. (2000). Ultraviolet detectors based on epitaxial ZnO films grown by MOCVD. J. Electron. Mater..

[29] Du G., Cui Y.G., Xia X.C., Li X.P., Zhu H.C., Zhang B.L., Zhang Y.T., Ma Y. (2007). Visual-infrared electroluminescence emission from ZnO/GaAs heterojunctions grown by metal-organic chemical vapor deposition. Appl. Phys. Lett..

[30] Li X.P., Zhang B.L., Dong X., Zhang Y.T., Xia X.C., Zhao W., Du G.T. (2009). Room temperature electroluminescence from ZnO/Si heterojunction devices grown by metal–organic chemical vapor deposition. J. Lumin..

[31] Zhang Y., Lee C.T. (2009). Site-controlled growth and field emission properties of ZnO nanorod arrays. J. Phys. Chem. C.

[32] Lee S.D., Kim Y.S., Yi M.S., Choi J.Y., Kim S.W. (2009). Morphology control and electroluminescence of ZnO nanorod/GaN heterojunctions prepared using aqueous solution. J. Phys. Chem. C.

[33] Bian J.M., Liu W.F., Sun J.C., Liang H.W. (2007). Synthesis and defect-related emission of ZnO based light emitting device with homo- and heterostructure. J. Mater. Process. Technol..

[34] Yuldashev S.U., Nusretov R.A., Khvan I.V., Yalishev V.S., Kang T.W. (2008). White light emission from ZnO/Zn_0:9_Mg_0:1_O heterostructures grown on Si substrates. Jpn. J. Appl. Phys..

[35] Dutta M, Basak D. (2008). p-ZnO/n-Si heterojunction: Sol-gel fabrication, photoresponse properties, and transport mechanism. Appl. Phys. Lett..

[36] Bhole M.P., Patil D.S. (2007). Deposition of non-polar a-axis nanocrystalline ZnO thin films for light emitting applications. Optoelectron. Adv. Mater.-Rapid commun..

[37] Lin Y.J., Wu P.H., Tsai C.L., Liu C.J., Lin Z.R., Chang H.C., Lee C.T. (2008). Effects of Mg incorporation on the optical properties of ZnO prepared by the sol-gel method. J. Appl. Phys..

[38] Lin Y.J., Wu P.H., Tsai C.L., Liu C.J., Lee C.T., Chang H.C., Lin Z.R., Jeng K.Y. (2008). Mechanisms of enhancing band-edge luminescence of Zn_1−x_Mg_x_O prepared by the sol–gel method. J. Phys. D Appl. Phys..

[39] Chuang R.W., Wu R.X., Lai L.W., Lee C.T. (2007). ZnO-on-GaN heterojunction light-emitting diode grown by vapor cooling condensation technique. Appl. Phys. Lett..

[40] Liu D.S., Wu C.C., Lee C.T. (2005). A transparent and conductive film prepared by RF magnetron cosputtering system at room temperature. Jpn. J. App. Phys..

[41] Ohtomo A., Kawasaki M., Koida T., Masubuchi K., Koinuma H., Sakurai Y., Yoshida Y., Yasuda T., Segawa Y. (1998). Mg_x_Zn_1-x_O as a II–VI widegap semiconductor alloy. Appl. Phys. Lett..

[42] Gruber T., Kirchner C., Kling R., Reuss F., Waag A. (2004). ZnMgO epilayers and ZnO–ZnMgO quantum wells for optoelectronic applications in the blue and UV spectral region. Appl. Phys. Lett..

[43] Shan F.K., Kim B.I., Liu G.X., Liu Z.F., Sohn J.Y., Lee W.J., Shin B.C., Yu Y.S. (2004). Blueshift of near band edge emission in Mg doped ZnO thin films and aging. J. Appl. Phys..

[44] Ryu Y.R., Lee T.S., Lubguban J.A., Corman A.B., White H.W., Leem J.H., Han M.S., Park Y.S., Youn C.J., Kim W.J. (2006). Wide-band gap oxide alloy: BeZnO. Appl. Phys. Lett..

[45] Ryu Y., Lee T.S., Lubguban J.A., White H.W., Kim B.J., Park Y.S., Youn C.J. (2006). Next generation of oxide photonic devices: ZnO-based ultraviolet light emitting diodes. Appl. Phys. Lett..

[46] Ma D.W., Ye Z.Z., Chen L.L. (2004). Dependence of structural and optical properties of Zn_1–x_Cd_x_O films on the Cd composition. Phys. Stat. Sol. A.

[47] Gruber T., Kirchner C., Kling R., Reuss F., Waag A., Bertram F., Forster D., Christen J., Schreck M. (2003). Optical and structural analysis of ZnCdO layers grown by metalorganic vapor-phase epitaxy. Appl. Phys. Lett..

[48] Dingle R. (1969). Luminescent transitions associated with divalent copper impurities and the green emission from semiconducting Zinc Oxide. Phys. Rev. Lett..

[49] Vanheusde K., Seager C.H., Warren W.L., Tallant D.R., Voigt J.A. (1996). Correlation between photoluminescence and oxygen vacancies in ZnO phosphors. Appl. Phys. Lett..

[50] Vanheusden K., Warren W.L., Seager C.H., Tallant D.R., Voigt J.A., Gnade B.E. (1996). Mechanisms behind green photoluminescence in ZnO phosphor powders. J. Appl. Phys..

[51] Kang H.S., Kang J.S., Kim J.W., Lee S.Y. (2003). Annealing effect on the property of ultraviolet and green emissions of ZnO thin films. J. Appl. Phys..

[52] Shan F.K., Liu G.X., Lee W.J., Shin B.C. (2007). The role of oxygen vacancies in epitaxial-deposited ZnO thin films. J. Appl. Phys..

[53] Reynolds D.C., Look D.C., Jogai B. (2001). Fine structure on the green band in ZnO. J. Appl. Phys..

[54] Heo Y.W., Norton D.P., Pearton S.J. (2005). Origin of green luminescence in ZnO thin film grown by molecular-beam epitaxy. J. Appl. Phys..

[55] Leiter F.H., Alves H.R., Hofstaetter A., Hofmann D.M., Meyer B.K. (2001). The oxygen vacancy as the origin of a green emission in undoped ZnO. Phys. Sta. Sol. B.

[56] Leiter F., Alves H., Pfisterer D., Romanov N.G., Hofmann D.M., Meyer B.K. (2003). Oxygen vacancies in ZnO. Physica B.

[57] Hofmann D.M., Pfisterer D., Sann J., Meyer B.K., Tena-Zaera R., Munoz-Sanjose V.T., Pensl Frank G. (2007). Properties of the oxygen vacancy in ZnO. Appl. Phys. A.

[58] Lai L.W., Yan J.T., Chen C.H., Lou L.R., Lee C.T. (2009). Nitrogen function of aluminum-nitride codoped ZnO films deposited using cosputter system. J. Mater. Res..

[59] Lin P.F., Ko C.Y., Lin W.T., Lee C.T. (2007). Effects of processing parameters on ultraviolet emission of In-doped ZnO nanodisks grown by carbothermal reduction. Mater. Lett..

[60] Kappers L.A., Gilliam O.R., Evans S.M., Halliburton L.E., Giles N.C. (2008). EPR and optical study of oxygen and zinc vacancies in electron-irradiated ZnO. Nucl. Instr. Meth. Phys. Res. B.

[61] Vlasenko L.S., Watkins G.D. (2005). Optical detection of electron paramagnetic resonance in room-temperature electron-irradiated ZnO. Phys. Rev. B.

[62] Oba F., Togo A., Tanaka I., Paier J., Kresse G. (2008). Defect energetics in ZnO: A hybrid Hartree-Fock density functional study. Phys. Rev. B.

[63] Korsunska N.O., Borkovska L.V., Bulakh B.M., Khomenkova L.Yu., Kushnirenko V.I., Markevich I.V. (2003). The influence of defect drift in external electric field on green luminescence of ZnO single crystals. J. Lumin..

[64] Lin B.X., Fu Z.X., Jia Y.B. (2001). Green luminescent center in undoped zinc oxide films deposited on silicon substrates. Appl. Phys. Lett..

[65] Hur T.B., Jeen G.S., Hwang Y.H., Kim H.Y. (2003). Photoluminescence of polycrystalline ZnO under different annealing conditions. J. Appl. Phys..

[66] Reshchikov M.A., Morkoc H., Nemeth B., Nause J., Xie J., Hertog B., Osinsky A. (2007). Luminescence properties of defects in ZnO. Physica B.

[67] Reshchikov M.A., Garbus J., Lopez G., Ruchala M., Nemeth B., Nause J. (2007). Acceptors in ZnO studied by photoluminescence. Res. Soc. Proc..

[68] Kohan A.F., Ceder G., Morgan D., Van de Walle C.G. (2000). First-principles study of native point defects in ZnO. Phys. Rev. B.

[69] Janotti A., Van de Walle C.G. (2005). Oxygen vacancies in ZnO. Appl. Phys. Lett..

[70] Patterson C.H. (2006). Role of defects in ferromagnetism in Zn_1−*x*_Co*_x_*O: A hybrid density-functional study. Phys. Rev. B.

[71] Hu J., Pan B.C. (2008). Electronic structures of defects in ZnO: Hybrid density functional studies. J. Chem. Phys..

[72] Janotti A., Van de Walle C.G. (2007). Native point defects in ZnO. Phys. Rev. B.

[73] Tomlins G.W., Routbort J.L., Mason T.O. (2000). Zinc self-diffusion, electrical properties, and defect structure of undoped single crystal zinc oxide. J. Appl. Phys..

[74] Kröger F.A. (1973). The Chemistry of Imperfect Crystals.

[75] Lany S., Zunger A. (2007). Dopability, intrinsic conductivity, and nonstoichiometry of transparent conducting oxides. Phys. Rev. Lett..

[76] Van de Walle C.G., Neugebauer J. (2004). First-principles calculations for defects and impurities: Applications to III-nitrides. J. Appl. Phys..

[77] Look D.C., Hemsky J.W., Sizelove J.R. (1999). Residual native shallow donor in ZnO. Phys. Rev. Lett..

[78] Lander J.J. (1957). Concentration of hydrogen and semi-conductivity in ZnO under hydrogen-ion bombardment. J. Phys. Chem. Solids.

[79] Janotti A., Van de Walle C.G. (2007). Hydrogen multicentre bonds. Nat. Mater..

[80] Lany S., Zunger A. (2005). Anion vacancies as a source of persistent photoconductivity in II-VI and chalcopyrite semiconductors. Phys. Rev. B.

[81] Smith J.M., Vehse W.E. (1970). ESR of electron irradiated ZnO confirmation of the F^+^ center. Phys. Lett. A.

[82] Soriano V., Galland D. (1976). Photosensitivity of the EPR spectrum of the F^+^ center in ZnO. Phys. Status Solidi B.

[83] Zhang Y.Z., Lu J.G., Ye Z.Z., He H.P., Zhu L.P., Zhao B.H., Wan L. (2008). Effects of growth temperature on Li–N dual-doped p-type ZnO thin films prepared by pulsed laser deposition. Appl. Surf. Sci..

[84] Lu J.G., Zhang Y.Z., Ye Z.Z., Zeng Y.J., He H.P., Zhu L.P., Huang J.Y., Wang L., Yuan J., Zhao B.H., Li X.H. (2006). Control of *p*- and *n*-type conductivities in Li-doped ZnO thin films. Appl. Phys. Lett..

[85] Zeng Y.J., Ye Z.Z., Xu W.Z., Li D.Y., Lu J.G., Zhu L.P., Zhao B.H. (2006). Dopant source choice for formation of *p*-type ZnO: Li acceptor. Appl. Phys. Lett..

[86] Wu J., Yang Y.T. (2008). Deposition of K-doped p type ZnO thin films on (0001) Al_2_O_3_ substrates. Mater. Lett..

[87] Wu J., Yang Y.T. (2008). Fabrication and characterization of K-H co-doped p-ZnO thin films. J. Comput. Theor. Nanosci..

[88] Kim J.B., Byun D., Ie S.Y., Park D.H., Choi W.K., Choi J.W., Angadi B. (2008). Cu-doped ZnO-based p-n hetero-junction light emitting diode. Semicond. Sci. Technol..

[89] Deng R., Zou Y.M., Tang H.G. (2008). Correlation between electrical, optical properties and Ag2+ centers of ZnO:Ag thin films. Physica B.

[90] Kang H.S., Ahn B.D., Kim J.H., Kim G.H., Lim S.H., Chang H.W., Lee S.Y. (2006). Structural, electrical, and optical properties of *p*-type ZnO thin films with Ag dopant. Appl. Phys. Lett..

[91] Duan L., Lin B.X., Zhang W.Y., Zhong S., Fu Z.X. (2006). Enhancement of ultraviolet emissions from ZnO films by Ag doping. Appl. Phys. Lett..

[92] Park C.H., Zhang S.B., Wei S.H. (2002). Origin of *p*-type doping difficulty in ZnO: The impurity perspective. Phys. Rev. B.

[93] Bhuvana K.P., Elanchezhiyan J., Gopalakrishnan N., Balasubramanian T. (2008). Codoped (AlN) and monodoped (Al) ZnO thin films grown by RF sputtering: A comparative study. Appl. Surf. Sci..

[94] Kim J.K., Lim J.W., Kim H.T., Kim S.H., Yun S.J. (2009). Fabrication of p-type ZnO thin films using rf-magnetron sputter deposition. Electrochem. Solid-State Lett..

[95] Lorenz M., Cao B.Q., Zimmermann G., Biehne G., Czekalla C., Frenzel H., Brandt M., von Wenckstern H., Grundmann M. (2009). Stable p-type ZnO:P nanowire/n-type ZnO:Ga film junctions reproducibly grown by two-step pulsed laser deposition. J. Vac. Sci. Technol. B.

[96] Hu G.X., Gong H. (2008). Unexpected influence of substrate temperature on the properties of P-doped ZnO. Acta Mater..

[97] Look D.C., Renlund G.M., Burgener II R.H., Sizelove J.R. (2004). As-doped p-type ZnO produced by an evaporation/sputtering process. Appl. Phys. Lett..

[98] Wang S.P., Shan C.X., Li B.H., Zhang J.Y., Yao B., Shen D.Z., Fan X.W. (2009). A facile route to arsenic-doped p-type ZnO films. J. Cryst. Growth.

[99] Zhang J.Y., Li P.J., Sun H., Shen X., Deng T.S., Zhu K.T., Zhang Q.F., Wu J.L. (2008). Ultraviolet electroluminescence from controlled arsenic-doped ZnO nanowire homojunctions. Appl. Phys. Lett..

[100] Xiu F.X., Yang Z., Mandalapu L.J., Zhao D.T., Liu J.L. (2005). Photoluminescence study of Sb-doped *p*-type ZnO films by molecular-beam epitaxy. Appl. Phys. Lett..

[101] Chu S., Lim J.H., Mandalapu L.J., Yang Z., Li L., Liu J.L. (2008). Sb-doped p-ZnO/Ga-doped n-ZnO homojunction ultraviolet light emitting diodes. Appl. Phys. Lett..

[102] Li B.S., Liu Y.C., Zhi Z.Z., Shen D.Z., Lu Y.M., Zhang J.Y., Fan X.W., Mu R.X., Henderson D.O. (2003). Optical properties and electrical characterization of p-type ZnO thin films prepared by thermally oxiding Zn_3_N_2_ thin films. J. Mater. Res..

[103] Du G.T., Ma Y., Zhang Y.T., Yang T.P. (2005). Preparation of intrinsic and N-doped *p*-type ZnO thin films by metalorganic vapor phase epitaxy. Appl. Phys. Lett..

[104] Xiao Z.Y., Liu Y.C., Zhang J.Y., Zhao D.X., Lu Y.M., Shen D.Z., Fan X.W. (2005). Electrical and structural properties of p-type ZnO:N thin films prepared by plasma enhanced chemical vapour deposition. Semicond. Sci. Technol..

[105] Zhao J.L., Li X.M., Bian J.M., Yu W.D., Zhang C.Y. (2005). Growth of nitrogen-doped p-type ZnO films by spray pyrolysis and their electrical and optical properties. J. Cryst. Growth.

[106] Lu J.G., Zhang Y.Z., Ye Z.Z., Zhu L.P., Wang L., Zhao B.H., Liang Q.L. (2006). Low-resistivity, stable *p*-type ZnO thin films realized using a Li–N dual-acceptor doping method. Appl. Phys. Lett..

[107] Lin C.C., Chen S.Y., Cheng S.Y., Lee H.Y. (2004). Properties of nitrogen-implanted p-type ZnO films grown on Si_3_N_4_/Si by radio-frequency magnetron sputtering. Appl. Phys. Lett..

[108] Nakano Y., Morikawa T., Ohwaki T., Taga Y. (2006). Electrical characterization of *p*-type N-doped ZnO films prepared by thermal oxidation of sputtered Zn_3_N_2_ films. Appl. Phys. Lett..

[109] Wang C., Ji Z.G., Liu K., Xiang Y., Ye Z.Z. (2003). p-Type ZnO thin films prepared by oxidation of Zn_3_N_2_ thin films deposited by DC magnetron sputtering. J. Cryst. Growth.

[110] Tu M.L., Su Y.K., Ma C.Y. (2006). Nitrogen-doped *p*-type ZnO films prepared from nitrogen gas radiofrequency magnetron sputtering. J. Appl. Phys..

[111] Yamamoto T., Katayama-Yoshida H. (1999). Solution using a codoping method to unipolarity for the fabrication of p-type ZnO. Jpn. J. Appl. Phys..

[112] Singh A.V., Mehra R.M., Wakahara A., Yoshida A. (2003). p-Type conduction in codoped ZnO thin films. J. Appl. Phys..

[113] Bian J.M., Li X.M., Gao X.D., Yu W.D., Chen L.D. (2004). Deposition and electrical properties of N–In codoped p-type ZnO films by ultrasonic spray pyrolysis. Appl. Phys. Lett..

[114] Zhuge F., Ye Z.Z., Zhu L.P., Lü J.G., Zhao B.H., Huang J.Y., Zhang Z.H., Wang L., Ji Z.G. (2004). Electrical and optical properties of Al–N co-doped p-type zinc oxide films. J. Cryst. Growth.

[115] Lu J.G., Zhu L.P., Ye Z.Z., Zhuge F., Zhao B.H., Huang J.Y., Wang L., Yuan J. (2005). p-type ZnO films by codoping of nitrogen and aluminum and ZnO-based p–n homojunctions. J. Cryst. Growth.

[116] Bhuvana K.P., Elanchezhiyan J., Gopalakrishnana N., Shinb B.C., Balasubramaniana T. (2009). Realization of p-type conduction in (ZnO)_1-x_(AlN)_x_ thin films grown by RF magnetron sputtering. J. Alloys Compd..

[117] Wang H., Ho H.P., Lo K.C., Cheah K.W. (2008). Preparation of p-type ZnO films with (N,Ga) Co-doping by MOVPE. Mater. Chem. Phys..

[118] Sun H., Zhang Q., Zhang J., Deng T., Wu J. (2008). Electroluminescence from ZnO nanowires with a p-ZnO film/n-ZnO nanowire homojunction. Appl. Phys. B.

[119] Cao Y.G., Miao L., Tanemura S., Tanemura M., Kuno Y., Hayashi Y. (2006). Low resistivity p-ZnO films fabricated by sol-gel spin coating. Appl. Phys. Lett..

[120] Lai L.W., Yan J.T., Chen C.H., Lou L.R., Lee C.T. (2009). Nitrogen function of aluminum-nitride codoped ZnO films deposited using cosputter system. J. Mater. Res..

[121] Schmidt-Mende L, MacManus-Driscoll J.L. (2007). ZnO-nanostructures, defects, and devices. Mater. Today.

[122] Lee W., Jeong M.C., Myoung J.M. (2004). Fabrication and application potential of ZnO nanowires grown on GaAs(002) substrates by metal–organic chemical vapour deposition. Nanotechnology.

[123] Yuan G.D., Zhang W.J., Jie J.S., Fan X., Zapien J.A., Leung Y.H., Luo L.B., Wang P.F., Lee C.S., Lee S.T. (2008). p-Type ZnO nanowire arrays. Nano Lett..

[124] Wu J.J., Liu S.C. (2002). Low-temperature growth of well-aligned ZnO nanorods by chemical vapor deposition. Adv. Mater..

[125] Tak Y., Park D., Yong K. (2006). Characterization of ZnO nanorod arrays fabricated on Si wafers using a low-temperature synthesis method. J. Vac. Sci. Technol. B.

[126] Zhang Y., Chen D.J., Lee C.T. (2007). Free exciton emission and dephasing in individual ZnO nanowires. Appl. Phys. Lett..

[127] Lu C.Y., Chang S.J., Chang S.P., Lee C.T., Kuo C.F., Chang H.M. (2006). Ultraviolet photodetectors with ZnO nanowires prepared on ZnO:Ga/glass templates. Appl. Phys. Lett..

[128] Fonoberov V.A., Balandin A.A. (2004). Origin of ultraviolet photoluminescence in ZnO quantum dots: Confined excitons versus surface-bound impurity exciton complexes. Appl. Phys. Lett..

[129] Perebeinos V., Tersoff J., Avouris Ph. (2005). Effect of exciton-phonon coupling in the calculated optical absorption of carbon nanotubes. Phys. Rev. Lett..

[130] Press D., Gotzinger S., Reitzenstein S., Hofmann C., Loffler A., Kamp M., Forchel A., Yamamoto Y. (2007). Photon antibunching from a single quantum-dot-microcavity system in the strong coupling regime. Phys. Rev. Lett..

[131] Devos A., Poinsotte F., Groenen J., Dehaese O., Bertru N., Ponchet A. (2007). Strong generation of coherent acoustic phonons in semiconductor quantum dots. Phys. Rev. Lett..

[132] Bahnemann D.W., Kormann C., Hoffmann M.R. (1987). Preparation and characterization of quantum size zinc oxide: A detailed spectroscopic study. J. Phys. Chem..

[133] Han X.H., Wang G.Z., Wang Q.T., Cao L., Liu R.B., Zou B.G., Hou J.G. (2005). Ultraviolet lasing and time-resolved photoluminescence of well-aligned ZnO nanorod arrays. Appl. Phys. Lett..

[134] Choi Y.S., Kang J.W., Hwang D.K., Park S.J. (2010). Recent advances in ZnO-based LEDs. IEEE Trans. Electron Dev..

[135] Jiao S.J., Zhang Z.Z., Lu Y.M., Shen D.Z., Yao B.J., Zhang Y., Li B.H., Zhao D.X., Fan X.W., Tang Z.K. (2006). ZnO *p*-*n* junction light-emitting diodes fabricated on sapphire substrates. Appl. Phys. Lett..

[136] Liu W., Gu S.L., Ye J.D., Zhu S.M., Liu S.M., Zhou X., Zhang R., Shi Y., Zheng Y.D., Hang Y., Zhang C.L. (2006). Blue-yellow ZnO homostructural light-emitting diode realized by metalorganic chemical vapor deposition technique. Appl. Phys. Lett..

[137] Wei Z.P., Lu Y.M., Shen D.Z., Zhang Z.Z., Yao B., Li B.H., Zhang J.Y., Zhao D.X., Fan X.W., Tang Z.K. (2007). Room temperature *p*-*n* ZnO blue-violet light-emitting diodes. Appl. Phys. Lett..

[138] Ye Z.Z., Lu J.G., Zhang Y.Z., Zeng Y.J., Chen L.L., Zhuge F., Yuan G.D., He H.P., Zhu L.P., Huang J.Y., Zhao B. H. (2007). ZnO light-emitting diodes fabricated on Si substrates with homobuffer layers. Appl. Phys. Lett..

[139] Du G.T., Liu W.F., Bian J.M., Hu L.Z., Liang H.W., Wang X.S., Liu A.M., Yang T.P. (2006). Room temperature defect related electroluminescence from ZnO homojunctions grown by ultrasonic spray pyrolysis. Appl. Phys. Lett..

[140] Yu Q.X., Xu B., Wu Q.H., Liao Y., Wang G.Z., Fang R.C., Lee H.Y., Lee C.T. (2003). Optical properties of ZnO/GaN heterostructure and its near-ultraviolet light-emitting diode. Appl. Phys. Lett..

[141] Rogers D.J., Teherani F.H., Yasan A., Minder K., Kung P., Razeghi M. (2006). Electroluminescence at 375 nm from a ZnO/GaN:Mg/*c*-Al_2_O_3_ heterojunction light emitting diode. Appl. Phys. Lett..

[142] Long H., Fang G., Huang H., Mo X., Xia W., Dong B., Meng X., Zhao X. (2009). Ultraviolet electroluminescence from ZnO/NiO-based heterojunction light-emitting diodes. Appl. Phys. Lett..

[143] Titkov I.E., Delimova L.A., Zubrilov A.S., Seredova N.V., Liniichuk I.A., Grekhov I.V. (2009). ZnO/GaN heterostructure for LED applications. J. Modern Optics.

[144] Bayram C., Teherani F.H., Rogers D.J., Razeghi1 M. (2008). A hybrid green light-emitting diode comprised of *n*-ZnO/(InGaN/GaN) multi-quantum-wells/*p*-GaN. Appl. Phys. Lett..

[145] Wang Y.L., Kim H.S., Norton D.P., Pearton S.J., Ren F. (2008). Dielectric passivation effects on ZnO light emitting diodes. Appl. Phys. Lett..

[146] Mandalapu L.J., Yang Z., Chu S., Liu J.L. (2008). Ultraviolet emission from Sb-doped *p*-type ZnO based heterojunction light-emitting diodes. Appl. Phys. Lett..

[147] Kim H.S., Lugo F., Pearton S.J., Norton D.P., Wang Y.L., Ren F. (2008). Phosphorus doped ZnO light emitting diodes fabricated via pulsed laser deposition. Appl. Phys. Lett..

[148] Ryu Y.R., Lubguban J.A., Lee T.S., White H.W., Jeong T.S., Youn C. J., Kim B.J. (2007). Excitonic ultraviolet lasing in ZnO-based light emitting devices. Appl. Phys. Lett..

[149] Chu S., Olmedo M., Yang Z., Kong J.Y., Liu J.L. (2008). Electrically pumped ultraviolet ZnO diode lasers on Si. Appl. Phys. Lett..

[150] Chen P.L., Ma X.Y., Yang D.R. (2006). Fairly pure ultraviolet electroluminescence from ZnO-based light-emitting devices. Appl. Phys. Lett..

[151] Ma X.Y., Chen P.L., Li D.S., Zhang Y.Y., Yang D.R. (2007). Electrically pumped ZnO film ultraviolet random lasers on silicon substrate. Appl. Phys. Lett..

[152] Yang Y., Sun X.W., Tay B.K., You G.F., Tan S.T., Teo K.L. (2008). A *p*-*n* homojunction ZnO nanorod light-emitting diode formed by As ion implantation. Appl. Phys. Lett..

[153] Jeong M.C., Oh B.Y., Ham M.H., Myounga J.M. (2006). Electroluminescence from ZnO nanowires in *n*-ZnO film/ZnO nanowire array/*p*-GaN film heterojunction light-emitting diodes. Appl. Phys. Lett..

[154] Bao J.M., Zimmler M.A., Capasso F., Wang X.W., Ren Z.F. (2006). Broadband ZnO single-nanowire light-emitting diode. Nano Lett..

[155] Ko1nenkamp R., Word R.C., Godinez M. (2005). Ultraviolet electroluminescence from ZnO/polymer heterojunction light-emitting diodes. Nano Lett..

[156] Zimmler M.A., Voss T., Ronning C., Capasso F. (2009). Exciton-related electroluminescence from ZnO nanowire light-emitting diodes. Appl. Phys. Lett..

[157] Leong E.S.P., Yu S.F. (2006). UV random lasing action in p-SiC(4H)/i-ZnO–SiO_2_ nanocomposite/n-ZnO:Al heterojunction diodes. Adv. Mater..

[158] Lee C.T., Lin Y.H., Lai L.W., Lou L.R. (2010). Mechanism investigation of p-i-n ZnO-based light-emitting diodes. IEEE Photo. Technol. Lett..

[159] Yan J.T., Chen C.H., Yen S.F., Lee C.T. (2010). Ultraviolet ZnO nanorod/*p*-GaN heterostructured light-emitting diodes. IEEE Photo. Technol. Lett..

[160] Lee C.T., Chou Y. H., Yan J.T., Lee H.Y. (2009). Light enhancement of Al nanoclusters embedded in Al-doped ZnO films of GaN-based light-emitting diodes. J. Vac. Sci. Technol. B.

